# The mRNA export adaptor Yra1 contributes to DNA double-strand break repair through its C-box domain

**DOI:** 10.1371/journal.pone.0206336

**Published:** 2019-04-05

**Authors:** Valentina Infantino, Evelina Tutucci, Noël Yeh Martin, Audrey Zihlmann, Varinia Garcia-Molinero, Géraldine Silvano, Benoit Palancade, Françoise Stutz

**Affiliations:** 1 Dept. of Cell Biology, 30 Quai E. Ansermet, University of Geneva, Geneva, Switzerland; 2 Department of Anatomy and Structural Biology, Albert Einstein College of Medicine, Bronx, NY, United States of America; 3 Centre for Integrative Biology (CIBIO), University of Trento, Povo, Trento, Italy; 4 Institut Jacques Monod, CNRS, UMR 7592, Univ Paris Diderot, Sorbonne Paris Cité, Paris, France; Universita degli Studi di Milano, ITALY

## Abstract

Yra1 is an mRNA export adaptor involved in mRNA biogenesis and export in *S*. *cerevisiae*. Yra1 overexpression was recently shown to promote accumulation of DNA:RNA hybrids favoring DNA double strand breaks (DSB), cell senescence and telomere shortening, via an unknown mechanism. Yra1 was also identified at an HO-induced DSB and Yra1 depletion causes defects in DSB repair. Previous work from our laboratory showed that Yra1 ubiquitination by Tom1 is important for mRNA export. Here, we found that Yra1 is also ubiquitinated by the SUMO-targeted ubiquitin ligases Slx5-Slx8 implicated in the interaction of irreparable DSB with nuclear pores. We further show that Yra1 binds an HO-induced irreparable DSB in a process dependent on resection. Importantly, a Yra1 mutant lacking the evolutionarily conserved C-box is not recruited to an HO-induced irreparable DSB and becomes lethal under DSB induction in a HO-cut reparable system. Together, the data provide evidence that Yra1 plays a crucial role in DSB repair via homologous recombination. While Yra1 sumoylation and/or ubiquitination are dispensable, the Yra1 C-box region is essential in this process.

## Introduction

Yra1 (Yeast RNA annealing protein 1) is an essential protein in *S*. *cerevisiae*, well characterized as an mRNA export adaptor involved in transcription elongation, 3’ processing, and finally mRNA export together with the Mex67/Mtr2 export receptor and the poly(A) binding protein Nab2 [1].

Yra1 is evolutionarily conserved from yeast to human and belongs to the RNA and Export Factor (REF) family of hnRNP-like proteins [2–5]. REF proteins include a conserved domain organization with a central RNP-motif containing an RNA binding domain (RBD) and two highly conserved N- and C-terminal boxes (N-box and C-box). These domains are separated by two variable regions (N-var and C-var), rich in positively charged amino acids that mediate interaction with RNAs and Mex67 [3, 6]. *yra1* mutants lacking the RBD, the N-terminal or C-terminal (N-box+N-var or C-box+C-var) regions are viable indicating functional redundancy in their RNA binding properties. However, at least one highly conserved N-box or C-box is required for viability as deletion of both is lethal [7].

While Mex67 and Nab2 are shuttling between the nucleus and cytoplasm, Yra1 is a strictly nuclear protein [3, 5]. Nuclear localization of Yra1 is important for mRNA export as mutants lacking the N-terminal nuclear localization signal (NLS) demonstrate nuclear accumulation of poly(A)+ RNA when examined by fluorescent *in situ* hybridization (FISH) [7]. Loss of the highly conserved Yra1 C-box (*yra1(1–210)* mutant) does not cause an obvious poly(A)+ mRNA export defect, but it is required for optimal growth [7]. This observation is consistent with the fact that the C-box does not play a major role in Mex67 or RNA binding and suggests that this highly conserved 16 amino acids sequence may be important for another aspect of Yra1 function.

Different layers of regulations have been shown to modulate Yra1 levels and function in mRNA biogenesis. We have previously shown that Yra1 ubiquitination by the E3 ligase Tom1 displaces Yra1 from messenger ribonucleoparticles (mRNPs) as a quality control signal for correctly processed mRNP prior to export into the cytoplasm [8]. Another important feature for Yra1 regulation is that the *YRA1* gene harbors the second largest intron (776 nt) in the *S*. *cerevisiae* genome. The presence of the *YRA1* intron is important to maintain optimal Yra1 protein levels through Yra1 auto-regulation at the level of splicing in a negative feedback mechanism [4]. Studies on the *YRA1* gene revealed that at least three elements contribute to optimal Yra1 autoregulation: a long first exon, a long intron, a weak branchpoint and an intact C-terminal domain [4]. The C-terminal domain was proposed to negatively regulate splicing provided that splicing efficiency was suboptimal.

Independent studies have also indicated that besides its function in mRNA biogenesis and export, Yra1 could contribute to DNA metabolism. It was initially proposed that Yra1 interacts with a subunit of the DNA polymerase δ and Dia2, an E3 ubiquitin ligase involved in DNA replication, genome stability and S phase checkpoint recovery [9, 10]. The C-box domain of Yra1 was suggested to be necessary for the recruitment of Dia2 at replication origins [10], establishing a potential functional link between Yra1 and DNA metabolism. Another report provided evidence that strong Yra1 overexpression causes transcription-associated hyper-recombination, a cell senescence-like phenotype and telomere shortening, probably by counteracting telomere replication since overexpressed Yra1 was located at the Y telomeric regions by ChIP-chip [11]. In the proposed model, Yra1 overexpression stabilizes R-loops, which contain DNA:RNA hybrids and displaced DNA strands, favoring conflicts between the replication fork and the RNA Pol II resulting in genome instability [11, 12]. Finally, a recent study based on ChAP-MS (chromatin affinity purification with mass spectrometry) identified Yra1 in association with a reparable double-strand break (DSB); moreover, a *yra1* DAmP (Decreased Abundance by mRNA Perturbation) hypomorph mutant showed sensitivity to DSB agents and global defects in DSB repair by pulse field gel electrophoresis [13].

DSBs can be repaired by two independent pathways: non-homologous end joining (NHEJ) that joins the DNA ends of the lesion in an error-prone process, and homologous recombination (HR), an error-free pathway used when homologous DNA sequences are available for the repair [14]. The genetic instability resulting from unrepaired DSBs leads to cell death [15]. The MRX complex (Mre11-Rad50-Xrs2) is implicated in the initial recognition of DSBs followed by NHEJ or HR depending on the cell cycle phase. In G1, the broken DNA ends are protected by the Ku70-Ku80 complex, which favors the action of the DNA ligase IV in joining the DNA ends via the NHEJ pathway [14]. In S and G2-M phases, the MRX complex together with Sae2 initiate resection by promoting endonucleolytic cleavage of the 5’ terminated DNA strands; this event is followed by extensive resection driven by the exonuclease Exo1 as well as by the helicase Sgs1 and the nuclease Dna2 forming the 3’ end ssDNA tails. The replication protein A (RPA) binds ssDNA and recruits proteins important for the DNA damage checkpoint that blocks the cell cycle, allowing DNA repair to occur [14]. A crucial component to start the HR process is the mediator Rad52 that displaces the RPA complexes to recruit the Rad51 recombinase on the 3’ end ssDNA tails. Once formed, the Rad51 nucleoprotein filament drives DNA strand invasion on the homologous template through different mechanisms that allow HR to occur [14, 16, 17]. The HR process has to be tightly regulated to avoid aberrant genomic rearrangements. On site sumoylation of HR proteins induced under DNA damage is pivotal to ensure efficient and optimal DSB repair [18–21]. SUMO-targeted E3 ubiquitin ligases (STUbL), such as the Slx5-Slx8 complex in yeast, have also been shown to contribute to the maintenance of genome stability, although their targets have not been systematically identified [22].

In this work, we show that Yra1 is sumoylated by the SUMO ligases Siz1 and Siz2, desumoylated by the SUMO protease Ulp1 and ubiquitinated by the SUMO-dependent E3 ligases Slx5-Slx8, which are important for genome integrity [22, 23]. Importantly, we find that Yra1 is recruited to DSBs in a resection-dependent process and identify the Yra1 C-box domain to be crucial for the binding and repair while Yra1 ubiquitination and/or sumoylation are not required in this process. Our results strengthen the importance of Yra1 in genome integrity and provide evidence for a critical role of Yra1 in DSB repair by homologous recombination.

## Materials and methods

### Yeast strains and plasmids

The strains and plasmids used in this study are listed in S1 and S2 Tables. Primers are listed in supplementary S3 Table.

The *YRA1* shuffled strains were obtained by transformation of the *YRA1* shuffle strain (*yra1*::*HIS3*, YCpLac33-URA3-*YRA1WT*, Cen) with YCpLac22-TRP centromeric plasmids encoding wild-type HA-tagged Yra1. The transformed strains were plated on 5-FOA to select against the *WT YRA1* URA3 plasmid. The cells able to grow on 5-FOA contain only the YCplac22-TRP1-*HA-YRA1 WT* plasmid (*YRA1* shuffled background). Single clones were analyzed for correct auxotrophic markers and checked for HA-Yra1 expression by Western blot with αHA antibodies.

The strains with integrated *HA-YRA1 WT* or *HA-yra1* mutant were obtained by transformation of the W303 Mat-a/α diploid strain or FSY5073 (GA-6844 HO irreparable system [24] with a fragment containing the HA-tagged wild-type or mutant *YRA1* sequences obtained by SmaI digestion of an engineered pUC18 construct. The pUC18 plasmids were obtained by Gibson assembly and contain a SmaI fragment consisting of the HA-tagged wild-type or mutant *YRA1* sequences preceded by the *YRA1* promoter and followed by the *YRA1* 3’ UTR, a selective marker (URA3 or HIS3) and an additional 100 pb of *YRA1* 3’ downstream sequences. Yeast transformants were plated on the relevant selective medium. Correct recombination and integration into the endogenous *YRA1* locus was checked by PCR with a forward primer complementary to a sequence -600bp upstream of the *YRA1* locus (OFS3118), not present in the plasmid sequence, and a reverse primer matching the HA-tag sequence present only in the plasmid-derived sequence (OFS3120). The W303 diploid strains containing the integrated *HA-YRA1 WT* or *HA-yra1* mutant sequences were sporulated on K-acetate agar plates for 3 days at 25°C and dissected. Single spores were analyzed for relevant auxotrophic markers; *HA-YRA1* integration was confirmed by PCR as described above and expression of HA-Yra1 proteins was verified by Western blot.

The deletion strains were generated by homologous recombination of a cassette containing an auxotrophic marker flanked by sequences adjacent to the gene to delete. The pUG73::*LEU2*, pAG25::natMX4 or pUG6::kanMX6 cassettes were amplified by PCR using 80 nucleotides long forward and reverse primers (20 nt complementary to the plasmid and 60 nt complementary to the target sequences). PCR products were transformed into the *YRA1* shuffle or *WT* W303 Mat-a/α diploid strains and correct insertion confirmed by PCR. The W303 Mat-a/α diploid strains containing the gene deletion were sporulated and single spores analyzed for auxotrophic markers. Haploid Mat-α *WT* W303 deletion mutants were crossed with haploid Mat-a strains containing integrated *HA-YRA1 WT* or mutant sequences obtained as described above. The diploid *yra1* double mutants were sporulated to obtain haploid *yra1* double mutants in W303 background. In the case of deletions in the *YRA1* shuffle, the *yra1* double mutants were obtained by plasmid shuffling as explained above.

The strains with integrated *HA-YRA1* in FSY6881 (NA17 strain with HO reparable system) [25] were obtained after four back crosses between the integrated *HA-YRA1 WT* or *HA*-*yra1(1–210)* and *HA-yra1allKR* mutants in W303 and the NA17 strain. The sporulation, dissection and analysis of the strains was performed as described above. The presence of the cassette KanMX::HO-cs at URA3 and KanMX::ClaI at LYS2 was checked by PCR followed by digestion with the restriction enzymes BamH1 (near the HO site) and ClaI.

### Media and culture conditions

If not specified, yeast strains were thawed on yeast extract-peptone-dextrose (YPD) plates and grown for two days at 25°C. Cells were pre-cultured in 5 ml of liquid YPD to reach an OD_600_ = 0.7–0.8 at 25°C and diluted into 100 ml YPD overnight culture to reach OD600 = 0.8/1 at 25°C in the morning.

For the protein stability assays using metabolic depletion of *GAL-HA-YRA1* in presence of the endogenous wild-type *YRA1* gene, cells expressing HA-Yra1 from the GAL promoter on a centromeric plasmid were grown over-night in selective medium containing 2% galactose. When reaching OD_600_ = 0.3, cells were shifted to selective medium containing 2% glucose to repress *GAL-HA-YRA1* and collected at time 0, 1h, 2h, 3h, 4h, 5h, 6h, and 7h following glucose addition.

To induce the HO endonuclease-mediated irreparable DSB, cells were grown over-night in SCLGg (SC lactate 2%/glycerol 2% containing 0.05% Glucose). Cells at OD = 0.4 were shifted to SCLGg medium containing 2% glucose for 2h (no cut induction) or to SCLGg medium containing 2% galactose to induce the HO endonuclease. Cells were collected at 30 minutes, 1h, 2h and 4h following galactose addition. To induce the HO endonuclease-mediated reparable DSB, cells were grown over-night in SCLGg (SC lactate 2%/glycerol 2% containing 0.05% Glucose). Exponentially growing cells were treated with 2% galactose to induce the HO endonuclease or not (control) for 2h. Serial dilutions of 200/100/50 cells were plated on SCLGg Glu 2%. In another related experiment, serial dilutions of exponentially growing cells in SCLGg medium were directly plated on SCLGg Gal 2% or SCLGg Gal 3%-Raf 1% to induce the HO cut, and on SCLGg Glu 2% to repress HO endonuclease expression.

### Spot test

Cells grown in YPD medium to stationary phase were diluted to OD_600_ = 1 and five 10-fold serial dilutions were prepared for spotting on agar plates. For each spot, 3μl were deposited on 2% glucose YPD plates in the presence or absence of drug (Zeocin 25 μg/ml, 50 μg/ml, and 100 μg/ml). Plates were incubated at 25°C, 30°C, 34°C or 37°C for 3 days.

Cells grown in SCLGg Leu- medium (SC lactate 2%/glycerol 2% containing 0.05% Glucose) to stationary phase were diluted to OD_600_ = 1 and five 10-fold serial dilutions were prepared for spotting on agar plates. For each spot, 3μl were deposited on 2% glucose SCLGg Leu-, 2% galactose SCLGg Leu-, 3% galactose/1% raffinose SCLGg Leu- plates. Plates were incubated at 25°C for 5 days.

### Protein extraction and western blotting

Cells were grown to OD_600_ = 1. Cell lysis was performed by adding 1 ml H_2_O with 150μl of Yex-lysis buffer (1.85M NaOH, 7.5% 2-mercaptoethanol) to the pellet of 5 ODs of cells and kept 10 minutes on ice. Proteins were precipitated by addition of 150μl of TCA 50% for 10 minutes on ice. The pellet was resuspended in 30μl of 1X sample buffer (1M Tris-HCl pH6.8, 8 M Urea, 20% SDS, 0.5M EDTA, 1% 2-mercaptoethanol, 0.05% bromophenol blue). Total protein extracts were fractioned on SDS-PAGE and examined by Western blotting with αHA (Enzo), αYra1 (Stutz laboratory), αPgk1 (Abcam), αRfa1, 2, 3 to detect RPA (kind gift from Vincent Géli), αGFP (Roche), αRad51 (Abcam), αRad53 total (EL7.E1), αRad53 phosphorylated form (F9.A1), αSUMO (Palancade laboratory) antibodies. For quantitative Western blot analyses, fluorescent secondary α-Mouse (IRDye 800CW) and α-Rabbit (IRDye 680RD) antibodies were used. The signals were revealed with the LYCOR instrument and quantified using LITE Software.

### Chromatin immunoprecipitation (ChIP) and quantitative real-time PCR

Cells grown to OD_600_ = 1 were cross-linked with 1.2% of formaldehyde (Molecular Biology grade Calbiochem^TM^) for 10 minutes at 25°C under continuous gentle agitation, quenched with 250mM of glycine (Sigma) for 5 min at 25°C and then on ice for at least 5 min, washed with PBS 1X and frozen at -20°C. Pellets of 100 ml cultures at OD_600_ = 1 were resuspended in 1ml of FA lysis buffer (10mM HEPES KOH pH 7.5, 140mM NaCl, 1mM EDTA pH 8, 1%Triton X-100, 0.1% sodium deoxycholate) containing a protease inhibitor cocktail (Complete tablets, Mini EDTA-free, Roche). Cells were mechanically broken with a magnalyser at 6500rpm for 30 seconds (4 times), and genomic DNA was sonicated for 20 cycles of 30 seconds ON/OFF in presence of 0.5% SDS added before the sonication step. Samples were centrifuged at 13000rpm for 15 min at 4°C, and chromatin (supernatant phase) was quantified by Bradford. For each IP, 1/10 of the total extract was kept as INPUT for final normalization. Chromatin extracts (500μg) were incubated at 4°C o/n with a specific antibody. In parallel, magnetic beads (Dynabeads Magnetic, Thermo Fisher Scientific) were incubated with BSA 5 mg/ml at 4°C o/n. The magnetic beads were washed twice with FA lysis buffer and resuspended with the same volume of FA lysis buffer containing a protease inhibitor cocktail (beads 50% v/v). The chromatin extracts with a specific antibody were incubated with 30μl of magnetic beads for 4h at 4°C on a rotating wheel. The magnetic beads were then washed twice with FA lysis buffer, twice with FA 500 (50mM HEPES KOH pH 7.5, 500mM NaCl, 1mM EDTA pH 8, 1%Triton X-100, 0.1% sodium deoxycholate), once with Buffer III (20mM Tris-HCl pH 8, 1mM EDTA pH 8, 250mM LiCl, 0.5% NP40, 0.5% sodium deoxycholate) and once with TE 1X (100mM Tris-HCl pH 8, 10mM EDTA pH 8). DNA was eluted with 200μl of elution buffer (50mM Tris-HCl pH 7.5, 1% SDS) at 65°C for 20 minutes. IP and INPUT DNAs were finally de-crosslinked with proteinase K (Roche) (0.4 μg/μl) for 2 hours at 42°C, and o/n at 65°C. The decrosslinked IP and INPUT DNAs were purified (Promega, Wizard Genomic DNA Purification Kit). IP and INPUT (2μl) were quantified by qPCR with SYBR Green PCR Master Mix (Applied Biosystems) using specific primers described in S3 Table. To check the HO cut induction, the corresponding locus was quantified by qPCR and the level was normalized to *SCR1*.

The following antibodies were used: a rabbit polyclonal αHA antibody (Enzo), a rabbit polyclonal αYra1 antibody and corresponding pre-immune (Stutz laboratory).

### Ubiquitination and Sumoylation assays

Ubiquitination and sumoylation assays were performed essentially as described [8, 26, 27] using cells transformed with a 2μ plasmid expressing His6-Ubi or His6-SUMO from a copper inducible promoter (*P*_*CUP1*_). Briefly Ubiquitin/SUMO expression was induced with 0.1 mM CuSO_4_ overnight or for 3h. Cell cultures (200 ml) at OD_600_ = 1 were collected adding TCA 5% for 20 minutes to allow protein precipitation. Cell pellets were washed twice with acetone 100%. Dry pellets were resuspended with 1ml of Guanidinium buffer (100 mM sodium phosphate at pH 8, 10 mM Tris-HCl, 6 M guanidinium, 10 mM imidazole, 0.2% Triton X-100, 10 mM NEM, complete protease inhibitor mix [Roche]) prior to cell disruption with glass beads in a magnalyser (6 cycles at 6500 rpm for 1 minute).

Cells lysates were spun at 13000 rpm for 20 min. Between 5–8 mg of protein from the supernatant was incubated with 100μl of Ni-NTA acid-agarose (Qiagen) for 2h at room temperature on a rotating wheel. Agarose beads were washed once with Guanidinium buffer and three times with Urea buffer (100 mM sodium phosphate at pH 6.8, 10 mM Tris-HCl, 8M urea, 20 mM imidazole, 0.2% Triton X-100, complete protease inhibitor mix [Roche]). His6-ubiquitinated and His6-SUMOylated proteins were eluted with 40 μl of Sample Buffer and boiled for 5 min at 95°C. 20 μl samples were analyzed by Western blot with the relevant antibodies: αHIS for ubiquitinated proteins, αSUMO for SUMOylated proteins, αHA or αYra1 for ubiquitinated or SUMOylated HA-Yra1 and Yra1 proteins. Input samples were also precipitated with TCA 5%, the pellets resuspended with Sample Buffer and boiled 5 min at 95°C to be analyzed by Western Blot with αHA for HA-Yra1, αYra1 for Yra1 and αPgk1 for Pgk1 as loading control.

### Poly(A)+ RNA FISH experiments

The FISH experiments on the *YRA1* shuffled strains deleted for various ubiquitin ligases were done essentially as described in [8], while the FISH experiments on the different *HA-YRA1* integrated strains were performed as described in [28]. In the latter case, images were acquired using an Olympus BX61 wide field epi-fluorescence microscope with a 100X/1.35NA UPlanApo objective. Samples were visualized using an X-Cite 120 PC lamp (EXFO) and the ORCA-R2 Digital CCD camera (Hamamatsu). Metamorph software (Molecular Devices) was used for acquisition. Z-sections were acquired at 200nm intervals over an optical range of 8.0 μm. In both cases Poly(A)^+^ mRNA in situ hybridization was performed with a Cy3-labeled oligo-dT_(50)_ probe.

### Colony forming unit assay (CFU)

In the reparable HO cut system, three serial dilutions (200/100/50) of exponentially growing cells in SCLGg medium were plated on SCLGg Gal 2% or SCLGg Gal 3%-Raf 1% or SCLGg Glu 2% and incubated at 25°C for 5 days. The percentage of colonies was determined as the relative number of Colony Forming Units (CFUs) in each strain plated on SCLGg Gal 2% or Gal 3%-Raf1% compared to the one plated on SCLGg Glu 2%. To normalize the variability in growth due to the different media condition, the CFUs of each strain transformed with pGal-HO endonuclease were normalized to the corresponding strain transformed with the empty vector (%CFU = (%CFU on SCLGg Gal 2% pGal-HO/EV)/ (% CFU on SCLGg Glu 2% pGal-HO/EV).

In the irreparable HO cut system, three serial dilutions of (10000/5000/2500) cells were plated on SCLGg Gal 2%-Raf 2% to induce the HO cut and three serial dilutions of 200/100/50 cells were plated on SCLGg-Raf 2% as control for the number of cells plated. Plates were incubated at 25°C for 8 days. The % of colonies was expressed as CFUs growing on SCLGg-Gal2%-Raf2% relative to the CFU growing on SCLGg-Raf 2% used as reference for the number of cells plated on SCLGg Gal 2%-Raf2%.

### Rad52 foci analysis

*YRA1 WT* and *yra1* mutants strains were transformed with the YCpLac111-*LEU2*-*RAD52*-YFP construct. Cells exponentially growing on selective medium were treated or not with Zeocin (100 μg/ml for 2h) and fixed with 4% PFA. Images were taken with the LSM700 microscope using laserline 405 nm for DAPI detection and laserline 514 nm for YFP, taking 8 z-stacks of 0.25 nm. Bright-field images were taken to define the cell cycle stage of the imaged cells. The Rad52-YFP foci were revealed and counted through the Z-stack images for each cell.

## Results

### Yra1 is modified by the SUMO-targeted E3 ubiquitin ligase Slx5-Slx8

Previous work from our laboratory showed that Yra1 ubiquitination by Tom1 elicits Yra1 dissociation from mRNPs, presumably in the context of the nuclear pore complex (NPC), allowing proper mRNP export into the cytoplasm [8]. Intriguingly, Yra1 ubiquitination is not fully abrogated in the *Δtom1* mutant, suggesting that other E3 ligases are involved in Yra1 regulation, possibly for other Yra1 functions. In view of the putative role of Yra1 in genome stability, we wondered whether this protein could be modified by SUMO-dependent ubiquitination.

**Fig 1:**
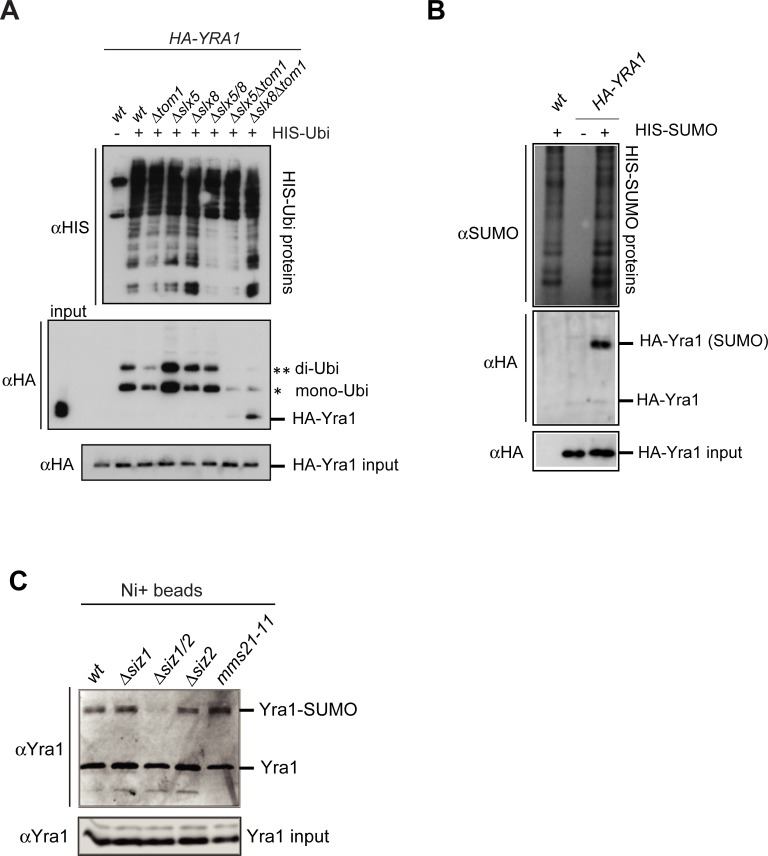
Yra1 is a sumoylated protein targeted for ubiquitination by the SUMO-dependent ubiquitin ligase Slx5-8. **(A)** Yra1 ubiquitination depends on the STUbL Slx5-Slx8 and Tom1. Ubiquitination assay of shuffled *HA-YRA1* in wild-type and in *Δtom1*, *Δslx8*, *Δslx5*, *Δslx8Δtom1*, *Δslx5Δtom1* and *Δslx8Δslx5* mutant backgrounds. His-Ubiquitinated proteins were affinity-purified and the Ubiquitinated forms of Yra1 detected by Western Blot with αHA antibodies. One representative experiment of 3 is shown. **(B)** Yra1 is sumoylated. Sumoylation assay in wild-type and *HA-YRA1* backgrounds. His-sumoylated proteins were affinity-purified and the sumoylated forms of Yra1 detected by Western Blot with αHA antibodies. One representative experiment of 3 is shown. **(C)** Yra1 is sumoylated by Siz1/Siz2. Yra1 sumoylation assay in wild-type as well as *Δsiz1*, *Δsiz1/siz2*, *Δsiz2* and *mms21-11* mutant backgrounds was performed as described above. His-sumoylated proteins were affinity-purified and the sumoylated forms of Yra1 detected by Western Blot with αYra1 antibodies. One representative experiment of 2 is shown.

We identified the SUMO-targeted E3 ubiquitin ligase (STUbL) complex Slx5-Slx8 to be responsible for Yra1 ubiquitination together with Tom1 (Fig 1).

The ubiquitination assay of HA-Yra1 in wild-type and in *Δtom1*, *Δslx5*, *Δslx8*, *Δslx5Δslx8*, *Δslx5Δtom1*, *Δslx8Δtom1* mutant backgrounds showed that the Yra1 ubiquitination detected in the *Δtom1* mutant was completely abrogated in the *Δslx5Δtom1* and *Δslx8Δtom1* double mutants (Fig 1A), indicating a role for both the Slx5-Slx8 and Tom1 E3 ligases in Yra1 regulation. Surprisingly, the ubiquitinated Yra1 levels are higher in *Δslx5*, but not in *Δslx8* and in *Δslx5Δslx8* mutants. This may reflect that Slx5 and Tom1 compete for Yra1 ubiquitination, and that loss of Slx5 thereby favors ubiquitination of Yra1 by Tom1, since the phenotype is lost in the *Δslx5Δslx8* double mutant. Because, the Slx5-Slx8 E3 ligase complex activity is stimulated by substrate sumoylation [29], and in view of the reported identification of Yra1 as potentially sumoylated in a proteome-wide study [30], we wondered whether Yra1 was itself modified by SUMO. We showed that Yra1 is indeed sumoylated (Fig 1B and 1C). Both Siz1 and Siz2 SUMO E3 ligases are involved in this modification as Yra1 sumoylation is fully abrogated in the *Δsiz1Δsiz2* double mutant background (Fig 1C). Furthermore, Yra1 is de-sumoylated by the SUMO protease Ulp1 as Yra1 sumoylation increased in the *ulp1* temperature-sensitive (*ts*) mutant (S1A Fig**).** These data support the hypothesis that Yra1 is regulated both by sumoylation and ubiquitination. In addition, HA-Yra1 ubiquitination was increased in the *ulp1 ts* mutant compared to a wild-type background, suggesting a possible stimulating effect of sumoylation on ubiquitination (S1B Fig). Conversely, sumoylation does not appear to depend on ubiquitination. Indeed, Yra1 can still be sumoylated in the *Δslx8Δtom1* strain in which ubiquitination of Yra1 is prevented; the result is not as clear in *Δslx5Δtom1* as the overall protein sumoylation is strongly reduced in this mutant (S1C Fig).

Known targets of Slx5-Slx8 are controlled by ubiquitin-dependent proteasomal degradation [31–35]. To define whether Yra1 ubiquitination by Slx5-Slx8 may target Yra1 to degradation, we used metabolic depletion to examine Yra1 turnover. Because *YRA1* is essential, an HA-tagged version of *YRA1* was expressed from a galactose-inducible promoter on a plasmid transformed into a strain expressing a wild-type *YRA1* gene. Switching cells from galactose to glucose-containing medium represses *GAL-HA-YRA1* gene expression and allows following the decay of the HA-Yra1 protein in different genetic backgrounds. Under metabolic glucose repression, HA-Yra1 has a half-life of 3.8h (S2A Fig). No significant stabilization of HA-Yra1 protein was detected in *Δslx8*, *Δslx5*, *Δtom1*, *Δslx8Δtom1*, *Δsiz1Δsiz2*, or the *ulp1 ts* mutant (S2B and S2C Fig), suggesting that sumoylation by Siz1-Siz2 and further ubiquitination by Slx5-Slx8 do not lead to Yra1 degradation by the proteasome.

We previously proposed that Yra1 regulation by Tom1 is linked to the function of Yra1 in mRNP export [1, 8]. Visualization of poly(A)+ RNA distribution by fluorescence in situ hybridization (FISH) in the *Δslx5* and *Δslx8* single mutants did not show any nuclear poly(A)+ RNA retention while the *Δslx5Δtom1* (32.3%) and *Δslx8Δtom1* (26%) double mutants had mRNA export defects comparable to the *Δtom1* mutant (30.8%) (S3A Fig). These observations suggest that Yra1 ubiquitination by Slx5-Slx8 may regulate a function of Yra1 distinct from mRNA export.

### Loss of the Yra1 C-box sensitizes the genome to DSBs

**Fig 2:**
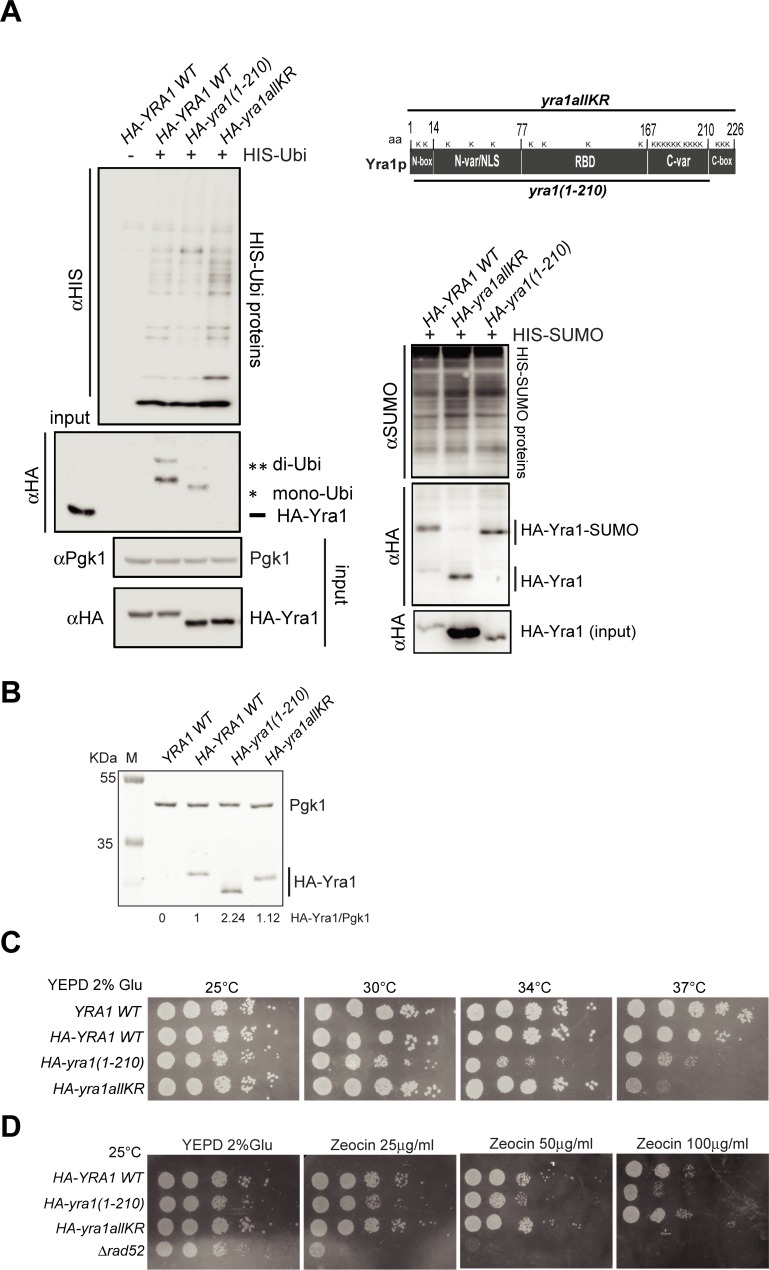
The Yra1 C-box, but not its ubiquitination and sumoylation, is important for genome stability. **(A)** Scheme of Yra1 mutants used in this study with corresponding ubiquitination and sumoylation assays. Left: Ubiquitination assay of shuffled *HA-YRA1 WT*, *HA-yra1(1–210)* and *HA-yra1allKR* mutants performed as described in Materials and Methods. Right: Sumoylation assay of shuffled *HA-YRA1 WT*, *HA-yra1allKR* and *HA-yra1(1–210)* mutants performed as described in Materials and Methods. One representative experiment of at least three is shown. Note that Yra1 is a very basic and charged protein and its non-modified forms tend to be retained on the Ni-NTA agarose beads. This is particularly striking with the HA-yra1allKR protein whose expression is increased in these strains. **(B)** Western Blot analysis of HA-Yra1 levels in integrated *HA-YRA1 WT*, *HA-yra1(1–210)*, *HA-yra1allKR*, was performed using an αHA antibody; an αPgk1 antibody was used as loading control. One representative Western blot is shown. Below: Western blot quantification showing the mean of the HA-Yra1/Pgk1 ratio of three experiments. **(C)** Spot test analysis of confluent cells at 25°C, 30°C, 34°C, 37°C of *YRA1 WT* (No Tag), integrated *HA-YRA1 WT*, *HA-yra1(1–210)*, and *HA-yra1allKR* strains on YEPD 2% Glucose. **(D)**. Spot test analysis on YEPD 2% Glu, Zeocin 25 μg/ml, Zeocin 50 μg/ml, Zeocin 100 μg/ml at 25°C of confluent cells of integrated *HA-YRA1 WT* and *HA-yra1* mutants as well as *Δrad52* strains.

Since our data indicate that Yra1 is modified by Slx5-Slx8, a STUbL important for genome stability [36], we examined whether the abrogation of Yra1 ubiquitination and sumoylation induces defects in genome integrity. For this purpose, we used the *HA-yra1allKR* mutant that cannot be ubiquitinated nor sumoylated since all the Lysines (K) are replaced by Arginines (R) (Fig 2A) [8].

We also used the *HA-yra1(1–210)* mutant which codes for a protein that is still ubiquitinated and sumoylated but lacks the highly conserved 16 C-terminal amino-acids (Fig 2A). Because Yra1 levels are maintained through splicing autoregulation, the intron was retained in both wild-type and mutant *HA-YRA1* constructs to limit the potential toxic effect of Yra1 overexpression [4, 11, 37–39]. Although the C-terminal domain has been implicated in splicing inhibition [4], the HA-yra1(1–210) protein is only mildly overexpressed compared to wild-type HA-Yra1; the HA-yra1allKR mutant is also slightly overexpressed indicating a potential role of post-translational modifications in splicing autoregulation (Fig 2A and 2B). Both mutants presents only a slight growth defect at 25°C but are thermosensitive as shown by spot test analysis at different temperatures (25°C, 30°C, 34°C and 37°C) (Fig 2C). Notably, the stronger thermosensitivity of the *HA-yra1allKR* mutant at 37°C may be linked to the overexpression of the HA-yra1allKR protein after 2h and 5h of growth at 37°C respectively of 1.75 and 1.98 fold increase (S3B Fig). Interestingly, additional spot test analyses in the presence of Zeocin indicated that the *HA-yra1(1–210)* but not the *HA-yra1allKR* mutant is sensitive to this genotoxic drug (Fig 2D). Importantly, the Zeocin treatment did not affect Yra1 ubiquitination and sumoylation (S4A Fig), nor Yra1 protein stability (S4B Fig); it also had no effect on the HA-Yra1 WT, HA-yra1(1–210) and HA-yra1allKR protein levels (S4C Fig). Finally, the lack of nuclear poly(A)+ RNA retention scored in these mutants in the conditions used in this study (25°C) was unchanged in the presence of Zeocin (S5A Fig). These observations indicate that the Yra1 C-box is important for genome stability in the presence of DNA double strand breaks (DSBs) while Yra1 ubiquitination and sumoylation are not. Importantly, this function of Yra1 is separable from its canonical role in mRNA export.

Double strand breaks cluster together in homologous recombination centers characterized by the co-localization with the repair factor Rad52. We monitored Rad52 foci formation in *yra1* mutants with and without Zeocin treatment to define whether they accumulate spontaneous DSBs *in vivo* and whether they are able to form Rad52 foci under DNA damage induction. The *yra1* mutants are able to form Rad52 foci under normal conditions at a frequency comparable to *YRA1 WT* cells; following Zeocin treatment, the number of Rad52 foci increases similarly in *the YRA1 WT* and *yra1* mutant cells (S5B Fig).

These results indicate that the Zeocin sensitivity of the *yra1(1–210)* mutant is not associated with impaired Rad52 foci formation.

### Yra1 is recruited to an irreparable DSB (HO cut)

**Fig 3:**
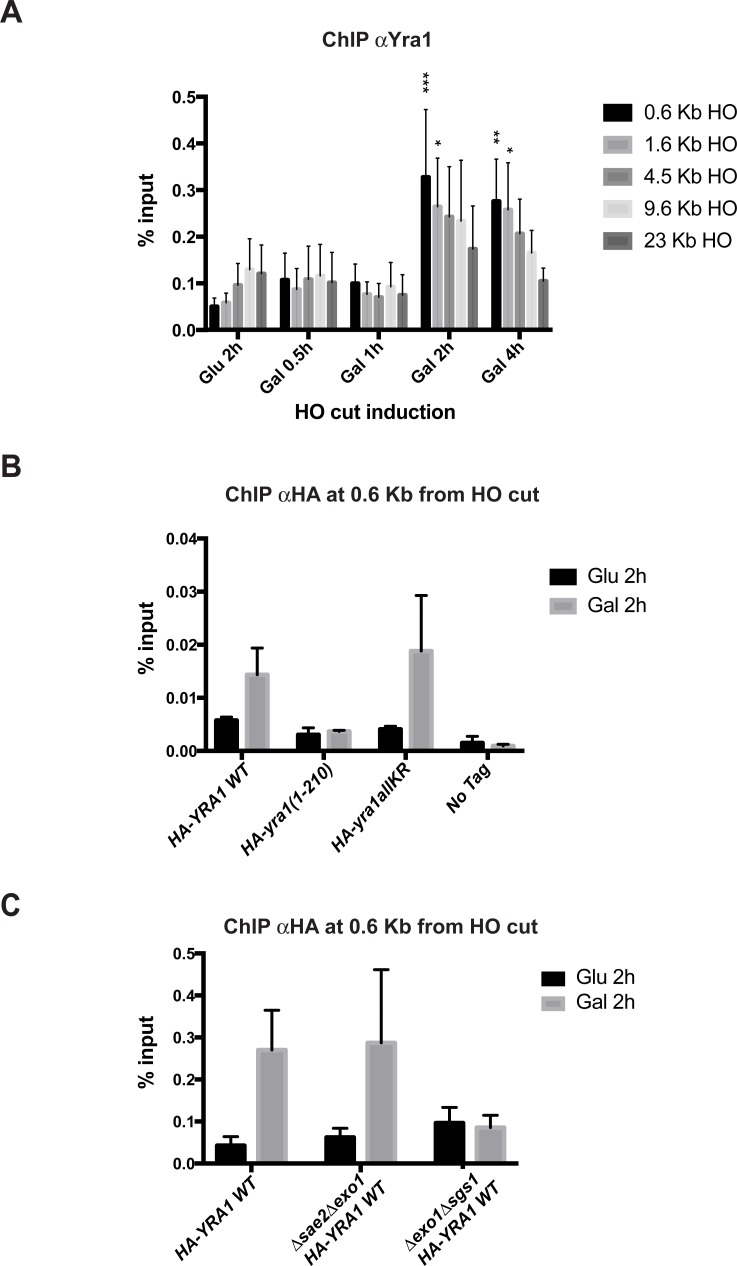
Yra1 is recruited to an irreparable DSB HO cut site. **(A)** Yra1 recruitment at the HO cut site was defined by ChIP with an αYra1 antibody after 0.5h, 1h, 2h and 4h of HO endonuclease induction with galactose using the GA6844 strain described in [22]. The 2h Glucose time point was used as no cut control. ChIP values are shown as percentage of input at 0.6Kb, 1.6Kb, 4.5Kb, 9.6Kb and 23Kb from the HO cut. The average of 6 experiments is shown with corresponding standard error of the mean. Two way ANOVA test was performed with multiple comparisons; P values < 0.05 (*), < 0.01 (**), < 0.001 (***) that refer to Glu 2h (no cut) are shown. **(B)** Yra1 mutants are differentially recruited to the irreparable HO cut site. ChIP using αHA antibody of HA-Yra1 WT, HA-yra1(1–210), HA-yra1allKR, Yra1 WT (no tag) at 0.6 Kb from the HO cut site after 2h of HO induction with Galactose using the strains with *HA-YRA1 WT* or mutants integrated in strain GA6844 described in [22]. The 2h Glucose time point was taken as no cut control. ChIP values are shown as percentage of input. The average of 3 independent experiments is shown with corresponding standard error of the mean. **(C)** Yra1 recruitment is dependent on extensive resection. HA-Yra1 WT recruitment at the HO cut site was defined by ChIP with an αHA antibody after 2h of HO endonuclease induction with galactose in *HA-YRA1 WT* combined with *Δsae2Δexo1* or *Δexo1Δsgs1*. The 2h Glucose time point was used as no cut control. ChIP values are shown as percentage of input at 0.6Kb from the HO cut. The average of 4 experiments is shown with corresponding standard error of the mean.

To obtain more direct evidence for a possible role of Yra1 in the DNA damage response pathway (DDR), we induced an irreparable DSB at the MAT locus using a galactose-inducible HO endonuclease as previously described [22] (S6A Fig). Consistent with the irreparable nature of the induced HO cut, these strains do not grow on galactose (S7A Fig). Yra1 recruitment at the HO cut, examined by ChIP with an αYra1 antibody, was significant at regions close to the DSB after 2h of HO induction (Fig 3A).

Considering that the efficiency of the cut is nearly 100% after 30’ of HO induction [22] (S8A Fig), the recruitment after 2h suggests it occurs following extensive resection.

To define whether the sensitivity to Zeocin of the *HA-yra1(1–210)* mutant may be due to its impaired recruitment to DSB loci, sequences encoding HA-tagged wild-type or mutant Yra1 (*HA-YRA1 WT*, *HA-yra1(1–210)* and *HA-yra1allKR*) were integrated into the irreparable HO DSB strain at the *YRA1* locus; the recruitment of these different HA-Yra1 proteins at the HO cut was examined by ChIP using αHA antibodies after 2h in galactose (Fig 3B), which induces efficient HO cleavage in both wild-type and mutant strains (S8B Fig). These experiments show that the HA-yra1allKR protein is recruited to the HO cut site to similar levels as the HA-Yra1 WT in Galactose (HO cut) (Fig 3B). In contrast, although in this experiment the HA-yra1(1–210) protein is expressed to slightly higher levels than HA-Yra1 WT (S7B and S7C Fig), its binding to the HO site does not increase in galactose, suggesting that the Yra1 C-terminal region is important for Yra1 recruitment to the DSB.

Since Yra1 is recruited to the HO cut 2h after Gal induction, once there has been extensive resection, we asked whether RPA binding to the HO cut might vary in the different *HA-yra1* mutants and whether Yra1 binding may depend on extensive resection. RPA association was not affected in the *HA-yra1* mutants despite the lack of HA-yra1(1–210) recruitment (S7D Fig), suggesting that RPA binding is probably not dependent on Yra1 recruitment. Importantly, HA-Yra1 binding to the HO cut was still present in the *Δsae2Δexo1* mutant (Fig 3C), in which the extensive resection is performed by the helicase Sgs1 [40, 41]; however HA-Yra1 recruitment was compromised in the *Δexo1Δsgs1* mutant (Fig 3C), in which the extensive resection is completely abrogated [40, 41]. The HA-Yra1 protein level as well as the HO cut efficiency in the *Δexo1Δsgs1* mutant are comparable to those measured in the WT background (S8C and S9A Figs). These observations support that Yra1 binding at the HO cut is dependent on extensive resection.

### The Yra1 C-terminal region is important for DSB repair (HO cut)

Since irreparable DSBs relocate to nuclear pores in G1/S phase [23] within 2h after cut induction [22], one possibility is that Yra1 recruitment to irreparable DSB is the consequence of HO cut re-localization to pores. To exclude this possibility, we took advantage of an HO cut reparable system (S6B Fig) [25], since DSB repair occurs within the nuclear interior [42, 43]. To define whether the *HA-yra1* mutants may be defective in DSB repair, the *HA-YRA1 WT*, *HA-yra1(1–210)* and *HA-yra1allKR* sequences were integrated at the *YRA1* locus of the reparable HO DSB strain and the percentage of cells surviving under HO cut induction was examined.

**Fig 4:**
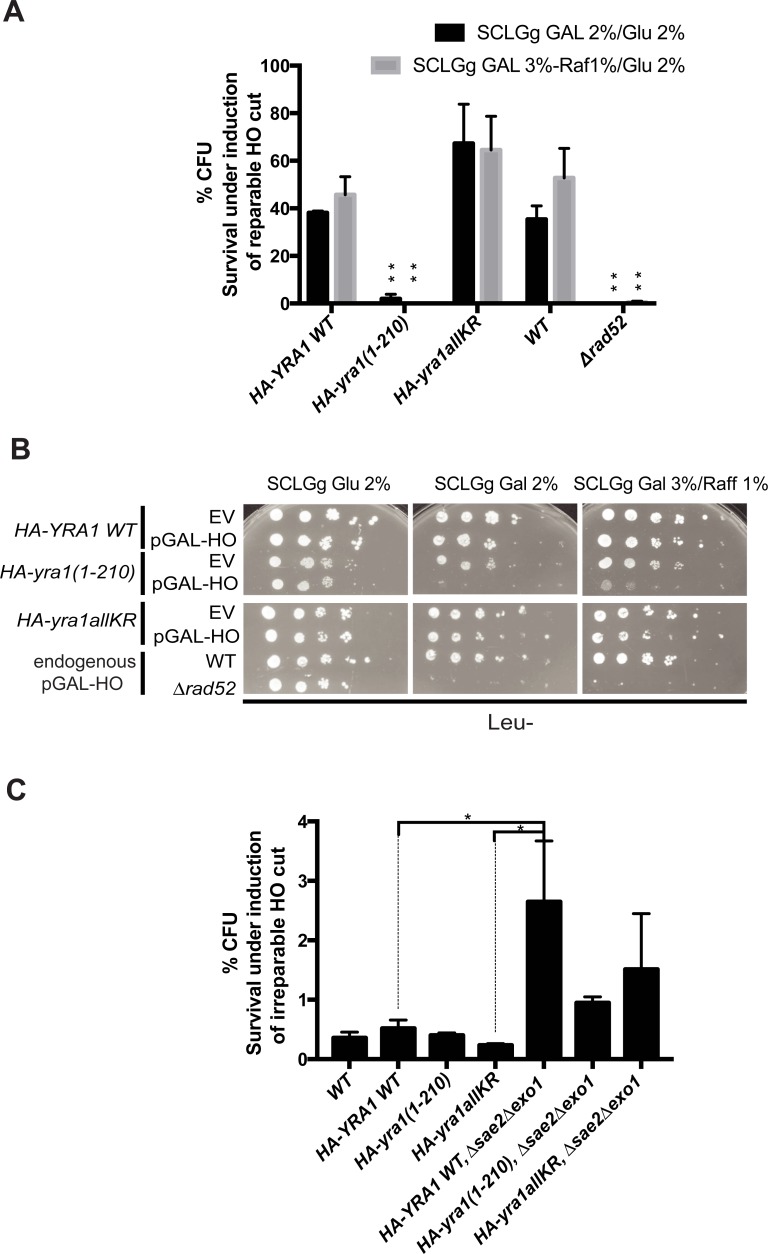
Survival under persistant induction of a reparable HO cut. **(A)** The Yra1 C-box is important for DSB repair. NA17 strains [25] containing integrated *HA-YRA1 WT* (WT), *HA-yra1(1–210)*, *HA-yra1allKR* were transformed with pGAL-HO endonuclease containing plasmid or Empty Vector, as well as No-Tag (NA17) and *Δrad52* strains containing endogenous pGAL-HO endonuclease were transformed with an Empty Vector. Diluted cells were plated on SCLGg Gal 2% or Gal 3%-Raf1% to constantly induce HO cut, and on SCLGg Glu 2% to repress HO endonuclease expression. The percentage colony forming units (CFUs) was determined as described in Materials and Methods. The average of 3 independent experiments for each condition SCLGg Gal 2%/ Glu 2% and SCLGg Gal 3%-Raf 1%/ Glu 2% is shown with corresponding standard error of the mean. One way ANOVA test was performed with multiple comparisons and P value < 0.001 (**) is shown on the graph referring to *HA-YRA1 WT*. **(B)** Yra1 C-box is important for DSB repair. Spot test analysis on Leu- SCLGg Glu 2%, SCLGg Gal 2%, SCLGg Gal 3%-Raf 1%, at 25°C of exponentially growing *HA-YRA1 WT* (WT), *HA-yra1(1–210)*, *HA-yra1allKR* (transformed with pGAL-HO endonuclease containing plasmid or Empty Vector); No-Tag and *Δrad52* strains (containing endogenous pGAL-HO endonuclease and transformed with an empty vector) served as controls. One representative experiment out of 3 is shown. **(C)** *yra1* mutant survival in the *Δsae2Δexo1* background under persistant induction of an irreparable HO cut. Dilutions of exponentially growing cells of GA6844 strain [22] containing integrated *HA-YRA1 WT* (WT), *HA-yra1(1–210)*, *HA-yra1allKR* combined or not with *Δsae2Δexo1* were plated on SCLGg Gal 2%-Raf2% to constantly induce the HO cut. Corresponding dilutions of cells were plated on SCLGg Raf2%. The percentage of Colony Forming Units (CFUs) was determined as described in Materials and Methods. The average of 2 independent experiments is shown with corresponding standard error of the mean. One way ANOVA test was performed with multiple comparisons and P value < 0.05 (*) is shown on the graph.

Three serial dilutions of exponentially growing cells were plated on galactose 2% or galactose 3%-raffinose 1% to induce the HO cut, and on glucose 2% to repress HO endonuclease expression. CFUs were counted as indication of cells able to repair the DSB in the *HA-YRA1 WT*, *HA-yra1(1–210)*, *HA-yra1allKR* strains transformed with a pGAL-HO endonuclease plasmid or Empty Vector; a *No-Tag* and a *Δrad52* strain transformed with the Empty Vector only were used as controls as these two strains contain an endogenous pGAL-HO endonuclease sequence (Fig 4A).

Interestingly, like the *Δrad52* control strain, the *HA-yra1(1–210)* mutant was not able to grow on galactose when the reparable HO cut is induced, indicating that the Yra1 C-box is important for DSB repair. This effect was confirmed by spot test analysis (Fig 4B). Notably, although both *HA-yra1* mutants have comparable cut efficiency after 2h in Galactose (S9B Fig), the *HA-yra1allKR* showed no growth defect under HO cut induction whether in the CFU assay or in the spot test, indicating that Yra1 ubiquitination and sumoylation are not required for DSB repair (Fig 4A and 4B).

DSBs can be repaired by NHEJ if they occur in the G1 phase and by HR if they occur in G2 and S phase [44]. During the G1 phase, the NHEJ pathway inhibits extensive resection crucial for the HR process [45]. We asked whether the *HA-yra1(1–210)* mutant that shows impaired DSB repair also has defects in NHEJ. To address this question we took advantage of the irreparable HO cut system (S6A Fig) since the HO cut cannot be repaired by HR and a low percentage of cells can survive thanks to the Break-Induced Replication (BIR) and the NHEJ pathways [46]. To define whether the *HA-yra1* mutants may be defective in NHEJ, the percentage of cells surviving under HO cut induction was examined in the *Δsae2Δexo1* background since cells lacking Sae2 promote the NHEJ pathway to repair the DSBs [47]. While the number of CFUs was clearly increased in the *Δsae2Δexo1* background compared to WT, the *HA-YRA1 WT*, *HA-yra1(1–210)* and *HA-yra1allKR* mutants showed a comparable number of colonies in both contexts, indicating that the *yra1* mutants do not have defects in the NHEJ pathway and BIR (Fig 4C).

Overall these observations support the view that Yra1 is important for DSB repair in a process dependent on the 16 amino acids C-terminal region and resection. Absence of this domain may result in the inability to repair HO cuts possibly because of the reduced capacity of Yra1 to interact with the DSB after extensive resection, a key step for the DSB repair by HR.

## Discussion

This study strengthens the importance of Yra1 in genome stability. In particular, our data provide evidence that the Yra1 C-terminal box is crucial for DSB repair. We have started to investigate the sensitivity of *yra1* mutants to DNA damage based on the observation that Yra1 is not only sumoylated by Siz1-Siz2 but also ubiquitinated by Slx5-Slx8, a SUMO-dependent E3 ligase important for genome stability (Fig 1). However, our data indicate that Yra1 ubiquitination and sumoylation are not important for DSB repair since the *HA-yra1allKR* mutant that completely abrogates Yra1 ubiquitination and sumoylation (Fig 2A**)** [8] does not display sensitivity to the DSB agent Zeocin (Fig 2D**)** nor any defect in DSB repair (Fig 4).

To investigate the effect of Yra1 on genome stability, we rather took advantage of the *HA-yra1(1–210)* mutant that lacks the Yra1 C-box domain (Fig 2). This domain does not interact with RNA [7] and the *HA-yra1(1–210)* mutant does not show any obvious mRNA export defect (S5A Fig). Interestingly, our data show that the *HA-yra1(1–210)* mutant is sensitive to the DSB inducing genotoxic agent Zeocin (Fig 2D), a phenotype that is not due to a defect in mRNA export in this condition (S5A Fig). In line with these results, it was recently published that the DAmP allele of *YRA1* is specifically sensitive to Zeocin [13]. Together, these observations suggest that lack of the Yra1 C-box either promotes DSBs or impairs DSB repair in a process independent of mRNA export activity.

A recent study revealed that Npl3, an RNA binding protein involved in mRNP biogenesis, contributes to DSB resection by ensuring efficient production of *EXO1* mRNA [48]. While Npl3 was proposed to have an indirect role in repair, our observations indicate that Yra1 is recruited to an irreparable DSB after 2h of cut induction and therefore extensive resection, consistent with a direct role of Yra1 in DSB repair (Fig 3). Accordingly, Yra1 binding at the HO cut is impaired in the *Δsae2Δexo1* mutant defective in the extensive resection event (Fig 3C). Importantly, the recruitment to an irreparable DSB does not depend on Yra1 ubiquitination and sumoylation but requires the conserved C-box, suggesting that this domain may be involved in repair, although it has no effect on RPA binding to the locus (Fig 3B and S7D Fig). The C-box could mediate Yra1 recruitment to DSBs by virtue of a direct interaction with resected DNA ends or DSB-associated proteins. However, we cannot fully exclude that Yra1 recruitment to irreparable DSBs may be the consequence of HO cut re-localization to the nuclear pore that occurs within 2h after cut induction [22].

Furthermore, we also examined whether the irreparable DSB can be repaired by alternative pathways such us Non Homologous End Joining (NHEJ) [14] or Break Induced Replication (BIR) [23] by inducing persistent irreparable HO cut in the *HA-yra1* mutants and plating cells on Galactose. To specifically favour the NHEJ pathway [47], the *HA-yra1* mutants were expressed in the *Δsae2Δexo1* background. The *YRA1 WT* and *yra1* mutants showed comparable colony formation ability both in a wild-type and *Δsae2Δexo1* background, indicating that the Yra1 C-box and Yra1 ubiquitination/sumoylation do not contribute to alternative repair pathways such as NHEJ (Fig 4C).

To directly address DSB repair efficiency in the *HA-yra1allKR* and *HA-yra1(1–210)* mutants, we used the HO reparable system described in [25]. Unfortunately, we were unable to observe significant recruitment of Yra1 to this type of DSB by ChIP, probably because the HO reparable system is more dynamic. However, an independent recent study identified Yra1 at an HO-induced reparable DSB using ChAP-MS (Chromatin Affinity Purification with mass spectrometry) [13]. These data indicate that Yra1 is recruited to the DSB locus also when the HO cut is located within the nucleus [49]. Thus, the observed Yra1 binding at the irreparable HO cut (Fig 3) may be specific rather than the indirect consequence of DSB relocalization to the nuclear periphery.

Besides detecting Yra1 at reparable DSBs, the recent study by Wang et al. [13] also shows that a Yra1 DAmP hypomorph mutant has a defect in global DSB repair following Zeocin treatment comparable to that observed in the absence of the key repair protein Rad52. As discussed by the authors, this global effect probably results from the reduced expression of Rad51 due to defective mRNA biogenesis and export activity in the presence of low levels of Yra1. The same study investigated the importance of Yra1 in the repair of a single HO cut using the Yra1 anchor away system. These experiments were unable to demonstrate a role for Yra1 in this process probably because the depletion by anchor away was not optimal. Since irreparable DSBs lead to cell death [14], we addressed the critical role of Yra1 in DSB repair by defining the repair efficiency of the *HA-yra1allKR* and *HA-yra1(1–210)* mutants based on survival following induction of the reparable HO cut (Fig 4A and 4B). Interestingly, while the *HA-yra1allKR* mutant has not effect, the *HA-yra1(1–210)* mutant exhibits very poor survival, comparable to that observed in *Δrad52* (Fig 4A and 4B). Since the *HA-yra1(1–210)* strain has no obvious mRNA export phenotype and exhibits normal Rad51 levels (S5A and S9C Figs), our data support the hypothesis that Yra1 may play a direct role in DSB repair by HR in a process that involves extensive resection and C-box-dependent recruitment of Yra1 to the resected damaged site (Fig 3). Since the resection at DSBs is paralleled by transcription inhibition of surrounding loci [50], it is likely that Yra1 binding to resected DNA ends is not linked to transcription. In conclusion, one possibility is that C-box-dependent Yra1 recruitment is important for repair by somehow favoring homologous recombination at the DSB. Eventhough we identified a link between the Yra1 C-box domain and the DBS repair process during extensive resection, our experiments did not reveal defects in the subsequent steps of HR such as RPA binding (S7D Fig), Rad51 levels (S9C Fig) and Rad52 foci formation (S5B Fig).

While our data show that Yra1 ubiquitination and sumoylation are not required for DSB repair, we cannot exclude that Yra1 modification by Slx5-Slx8 may facilitate relocalization of irreparable DSBs to nuclear pores [22, 23]. The physiological relevance of irreparable DSB relocation to the nuclear periphery is still not fully clear. It has been speculated that it leads to proteasomal degradation of DSB-bound proteins targeted by the STUbL Slx5-Slx8 [22] to induce alternative repair pathways such us Break Induced Replication [23]. In that respect, our data show that ubiquitination and sumoylation do not lead to Yra1 degradation (S2 Fig). Furthermore, Yra1 ubiquitination and sumoylation are not required for cell growth after irreparable DSB induction suggesting that they are not important for non-canonical repair and NHEJ (Fig 4C).

In summary, this work indicates that at physiological expression levels, Yra1 is beneficial for genome stability by facilitating the repair of DSBs by HR in a C-box-dependent and sumoylation/ubiquitination-independent manner. Future studies should address how Yra1 recruitment to DSBs may contribute to repair through homologous recombination.

## Supporting information

S1 Fig Yra1 sumoylation promotes its ubiquitination. **(A)** Yra1 is de-SUMOylated by Ulp1.
Sumoylation assay in wild-type and *ulp1* temperature-sensitive (ts) mutant as described in Materials and Methods. One representative experiment out of 3 is shown.
**(B)** Yra1 ubiquitination increases in the *ulp1* ts mutant.
Ubiquitination assay in wild-type and *ulp1* ts mutant as described in Materials and Methods. One representative experiment out of 3 is shown.
**(C)** Yra1 sumoylation in the *Δslx5-8*, *Δslx5Δtom1*, *Δslx8Δtom1* mutants.
Sumoylation assay in wild-type, *Δslx5*-8, *Δslx5Δtom1* and *Δslx8Δtom*1 mutants as described in Materials and Methods. One representative experiment out of 3 is shown.
(TIF)

S2 Fig Ubiquitination by Slx5-Slx8 does not affect Yra1 half-life. **(A)** Yra1 half-life is 3.8 h when using a metabolic Gal depletion assay. Protein stability assay using metabolic depletion of *GAL-HA-YRA1* in the presence of the endogenous wild-type *YRA1* gene was performed as described in Materials and Metods. HA-Yra1 protein levels were quantified by Western blot and normalized to Pgk1. The average of 2 independent experiments is shown. **(B)**, **(C)** Yra1 stability does not change in the absence of E3 ligases and SUMO protease Ulp1. Protein stability assay using metabolic depletion of *GAL-HA-YRA1* in the *YRA1* WT shuffle background combined with *Δslx8*, *Δslx5*, *Δtom1*, *Δslx8Δtom*1, in WT (W303) and *Δsiz1Δsiz2* at 25°C as well as in *ulp1 ts* and WT (W303) at 34°C. Western Blot analysis (B) and relative quantification (C) were performed as described in Materials and Methods. The average of at least 2 independent experiments (N2) is shown. Two way ANOVA statistical test with multiple comparisons did not show any statistically significant difference (n.s) between different yeast strains at the same time points.
(TIF)

S3 Fig The *Δslx5*, *Δslx8* and *yra1* mutants show no mRNA export defect by FISH analysis. **(A)** Fluorescent *in situ* hybridization (FISH) analysis of poly(A)+ RNA localization using oligo(dT) probes on shuffled *HA-YRA1 WT* in *WT*, *Δtom1*, *Δslx8*, *Δslx5*, *Δslx8Δtom1*, *Δslx5Δtom1* background and *mex67-5* cells as control for mRNA export defect. The percent of cells showing poly(A)+ RNA accumulation in the nucleus is indicated in each panel. DAPI stains the cell nucleus. **B)** Left: Western Blot analysis of *HA-Yra1 WT*, *HA-yra1(1–210)*, *HA-Yra1allKR* mutants grown at 25°C until exponential phase (37°C 0h) and shifted to 37°C for 2h and 5h. Right: graph representing the ratio HA-Yra1/Pgk1 of three independent experiments performed as described in Fig 2.
(TIF)

S4 Fig Yra1 is not affected by Zeocin treatment. **(A)** Yra1 ubiquitination and sumoylation do not change under Zeocin treatment. Exponentially growing cells were treated with Zeocin (100 μg/ml) for 2h and processed for the Ubiquitination (left) and Sumoylation (right) assays as described in Materials and Methods. Rad53 phosphorylation under Zeocin treatment was revealed by Western Blot using the EL7.E1 antibody against Rad53 total protein. (**B)** Yra1 stability does not change under Zeocin treatment. Protein stability assay using metabolic depletion of *GAL-HA-YRA1* was performed as described in Materials and Methods taking time points before (Gal O.N.) or 3h and 6h after adding Glucose. The Zeocin treatment (100 μg/ml) was started 2h before each sample collection. Rad53 phosphorylation under Zeocin was revealed by Western Blot using the EL7.E1 antibody against Rad53 total protein. Western Blot analysis of HA-Yra1 was performed as described in Materials and Methods. Quantification of the mean HA-Yra1/Pgk1 ratio of two experiments is indicated below. **(C)** Yra1 protein levels do not change under Zeocin treatment in the *HA-YRA1 WT*, *HA-yra1(1–210) and HA-yra1allKR* mutants. Exponentially growing cells were treated or not with Zeocin for 2h (100 μg/ml). Western blot analysis was performed as described in Materials and Methods. Quantification of the mean HA-Yra1/Pgk1 ratio of two experiments is indicated below. Rad53 phosphorylation under Zeocin treatment was revealed by Western Blot using the F9.A1 antibody against the Rad53 phosphorylated protein.
(TIF)

S5 Fig **(A) *HA-YRA1 WT*, *HA-yra1(1–210) and HA-yra1allKR* have no mRNA export defect at 25°C and under Zeocin treatment.** Fluorescent in situ hybridization (FISH) analysis of poly(A)+ RNA localization using oligo(dT) probes of integrated *HA-YRA1* WT, *HA-yra1(1–210)*, *HA-yra1allKR* and *mex67-5* cells. Cells were grown exponentially in YEPD 2% Glu at 25°C and treated for 2h with Zeocin (100 μg/ml). The *mex67-5* ts mutant was grown for an additional 1h at 37°C. One representative image of nuclear staining (DAPI), oligo-dT Cy3 (poly(A)+ RNA), and DIC is shown for each strain analyzed. The percent of cells showing poly(A)+ RNA accumulation in the nucleus is indicated on each panel.
**(B) *HA-YRA1 WT*, *HA-yra1(1–210) and HA-yra1allKR* are able to form Rad52 foci.** Rad52 foci were quantified after Zeocin treatment (2h 100 μg/ml) or not (Not Treated, NT) in the indicated strains as well as in *Δnup60* used as a positive control for Rad52 foci accumulation. One representative Z stack of YFP (Rad52-YFP), DAPI (nuclear staining) and Transmission Light (TL) is shown for each strain analyzed. The percent of cells with Rad52 foci is indicated in each panel.
(TIF)

S6 Fig Gal-induced HO-mediated irreparable and reparable DSB systems. **(A)** Scheme showing the Gal-induced HO-mediated irreparable DSB described in [24]. The HO endonuclease is expressed in the presence of Galactose, inducing the HO cut at the Mat locus that cannot be repaired because of the deletion of *HML* and *HMR*. **(B)** Scheme showing the Gal-induced HO-mediated reparable DSB described in [25]. The HO endonuclease is expressed in the presence of Galactose, inducing the HO cut at the KanMx cassette next to the *URA3* locus. The repair of the DSB at the HO cut is possible by HR thanks to the KanMX cassette at the *LYS2* locus. If this occurs, the repair will result in an HO insensitive KanMX cassette at the *URA3* locus as well as the loss of the short unique sequence surrounding the initial HO cut site.
(TIF)

S7 Fig Growth phenotypes and protein levels in the irreparable HO-cut *HA-YRA1 WT* and *HA*-*yra1* mutant strains. **(A)** Spot test analysis on plates containing SCLGg Glu 2% and SCLGg Gal 2% of confluent cultures of integrated *HA-YRA1 WT* and *HA*-*yra1* mutants containing the HO irreparable DSB. A *YRA1* WT strain without any galactose-inducible irreparable HO cut is shown as control. **(B)** Protein levels of HA-Yra1 WT, HA-yra1(1–210) and HA-yra1allKR expressed from copies integrated into the GA-6844 strain [22] after 2h in Glucose or Galactose to induce the irreparable HO cut. The levels of WT or mutant HA-Yra1 proteins remain quite constant between the different time points Glu 2h and Gal (0.5h, 1h, 2h). Values of HA-Yra1/Pgk1 are shown below the blot. One representative Western Blot is shown. **(C)** Quantification of the Western blot. The average of 3 independent experiments is shown with corresponding standard error of the mean. **(D)** RPA recruitment to the HO cut site in *yra1* mutants. ChIP using αRPA antibody of *HA-YRA1 WT* (WT), *HA-yra1(1–210)* and *HA-yra1allKR*, at 0.6 Kb from the HO cut site after 2h of HO induction with Galactose. The 2h Glucose time point was taken as no cut control. ChIP values are shown as percentage of input. The average of 3 independent experiments is shown with corresponding standard error of the mean.
(TIF)

S8 Fig *yra1*, *Δsae2Δexo1* and *Δexo1Δsgs1* mutants have comparable HO cut efficiency. **(A)** Analysis of HO cut site levels in the GA6844 strain described in [22] after 0.5h, 1h, 2h and 4h of HO endonuclease induction with galactose. The HO cut genomic locus was quantified by qPCR and the level was normalized to *SCR1*. The average of 6 independent experiments is shown with corresponding standard error of the mean. **(B)** Analysis of HO cut site levels in the *HA-YRA1 WT* and *HA-yra1* mutants integrated in GA6844 strain described in [22] after 2h of HO endonuclease induction with galactose or 2h in Glucose (no HO induction). The average of 3 independent experiments is shown with corresponding standard error of the mean. **(C)** Analysis of HO cut site levels in the *HA-YRA1 WT* and *Δsae2Δexo1*, *Δexo1Δsgs1* mutants. The average of 4 independent experiments is shown with corresponding standard error of the mean.
(TIF)

S9 Fig **(A)** Left: Western Blot analysis related to Fig 3C of HA-Yra1 in WT, *Δsae2Δexo1*, *Δexo1Δsgs1* mutants and performed as described in Materials and Methods. Right: Quantification and average of 4 experiments with corresponding standard error of the mean.
**(B)** Analysis of HO cut site levels in *HA-YRA1 WT* (WT), *HA-yra1(1–210)*, *HA*-*yra1allKR* and No-Tag strains treated with Galactose 2% (cut induction) or not (control) for 2h. The average of 2 independent experiments is shown with corresponding standard error of the mean. **(C)** Levels of Rad51 in *HA-YRA1 WT* and *HA-yra1* mutant strains analyzed by Western blot. Right: Western blot quantification of the ratio of Rad51/Pgk1 of three independent experiments with relative standard error of the mean.
(TIF)

S1 Table Strains used in this study. (DOCX)

S2 Table Plasmids used in this study. (DOCX)

S3 Table Primers used in this study. (DOCX)
