# Supplementary figures and images for: The mRNA export adaptor Yra1 contributes to DNA double-strand break repair through its C-box domain
Source: PLoS One. 2019 Apr 5;14(4):e0206336. doi: 10.1371/journal.pone.0206336 (PMC6450643; doi:10.1371/journal.pone.0206336)

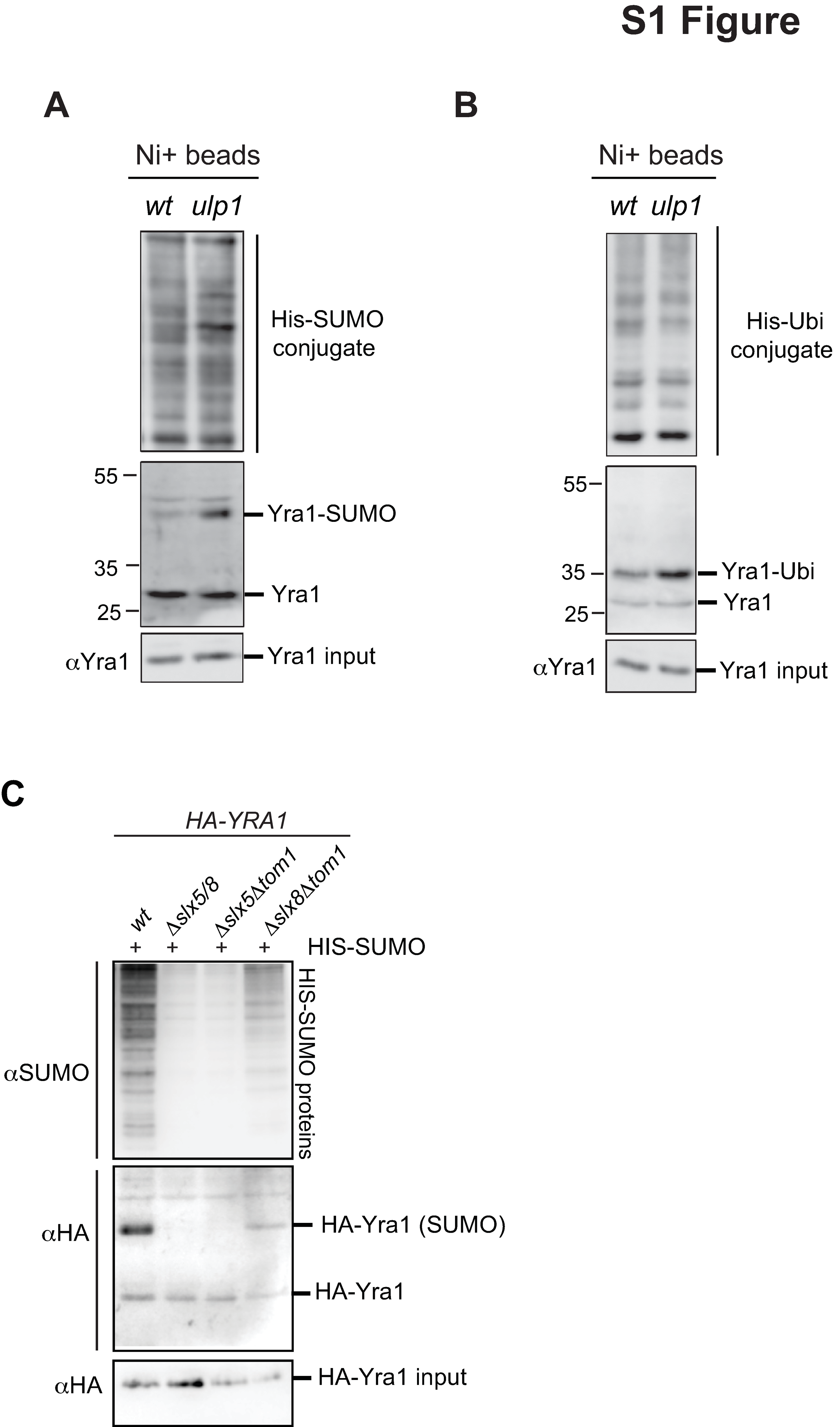

Supplement: S1 Fig — (A) Yra1 is de-SUMOylated by Ulp1. Sumoylation assay in wild-type and ulp1 temperature-sensitive (ts) mutant as described in Materials and Methods. One representative experiment out of 3 is shown. (B) Yra1 ubiquitination increases in the ulp1 ts mutant. Ubiquitination assay in wild-type and ulp1 ts mutant as described in Materials and Methods. One representative experiment out of 3 is shown. (C) Yra1 sumoylation in the Δslx5-8, Δslx5Δtom1, Δslx8Δtom1 mutants. Sumoylation assay in wild-type, Δslx5-8, Δslx5Δtom1 and Δslx8Δtom1 mutants as described in Materials and Methods. One representative experiment out of 3 is shown. (TIF) [file pone.0206336.s001.tif]

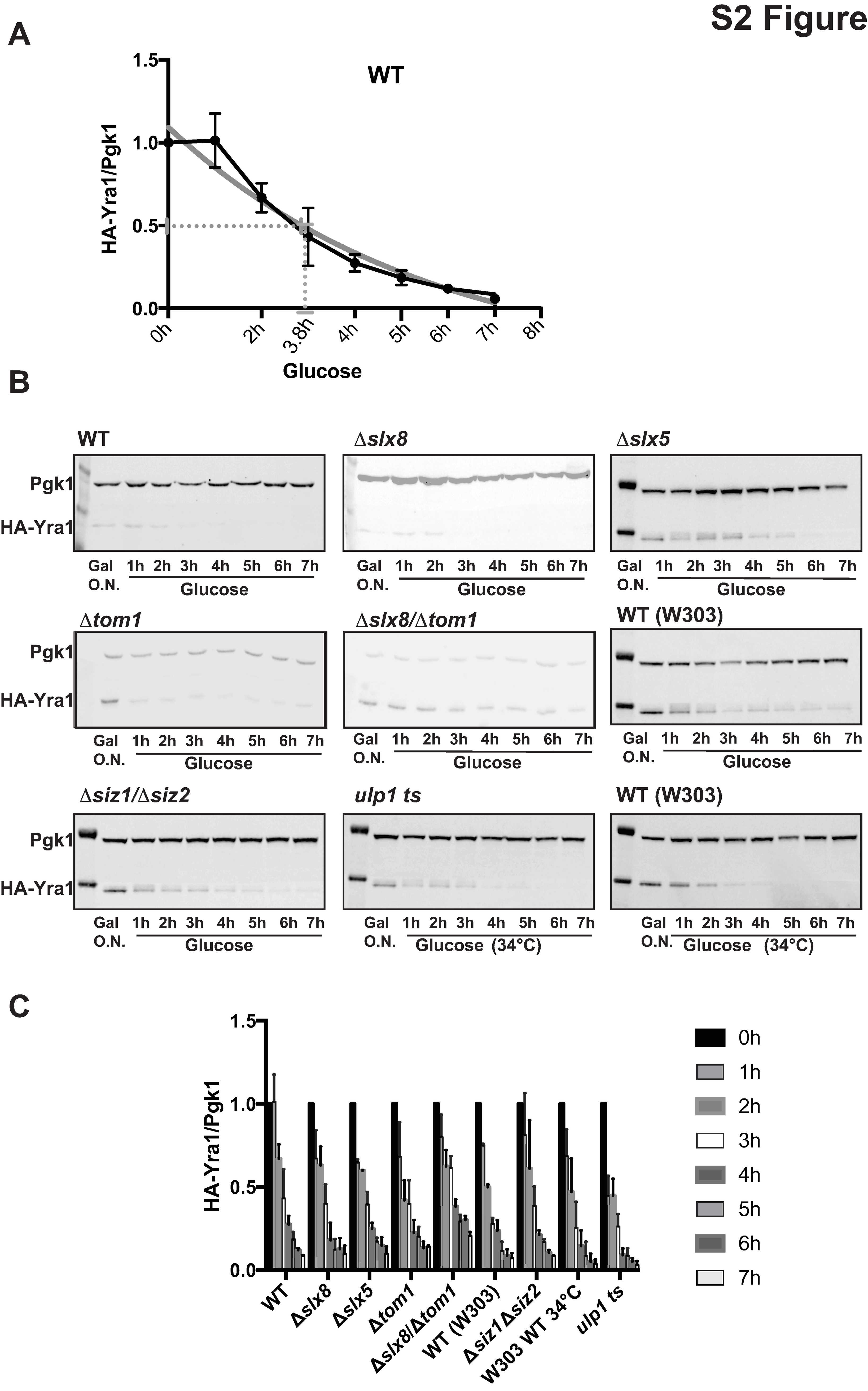

Supplement: S2 Fig — (A) Yra1 half-life is 3.8 h when using a metabolic Gal depletion assay. Protein stability assay using metabolic depletion of GAL-HA-YRA1 in the presence of the endogenous wild-type YRA1 gene was performed as described in Materials and Metods. HA-Yra1 protein levels were quantified by Western blot and normalized to Pgk1. The average of 2 independent experiments is shown. (B), (C) Yra1 stability does not change in the absence of E3 ligases and SUMO protease Ulp1. Protein stability assay using metabolic depletion of GAL-HA-YRA1 in the YRA1 WT shuffle background combined with Δslx8, Δslx5, Δtom1, Δslx8Δtom1, in WT (W303) and Δsiz1Δsiz2 at 25°C as well as in ulp1 ts and WT (W303) at 34°C. Western Blot analysis (B) and relative quantification (C) were performed as described in Materials and Methods. The average of at least 2 independent experiments (N2) is shown. Two way ANOVA statistical test with multiple comparisons did not show any statistically significant difference (n.s) between different yeast strains at the same time points. (TIF) [file pone.0206336.s002.tif]

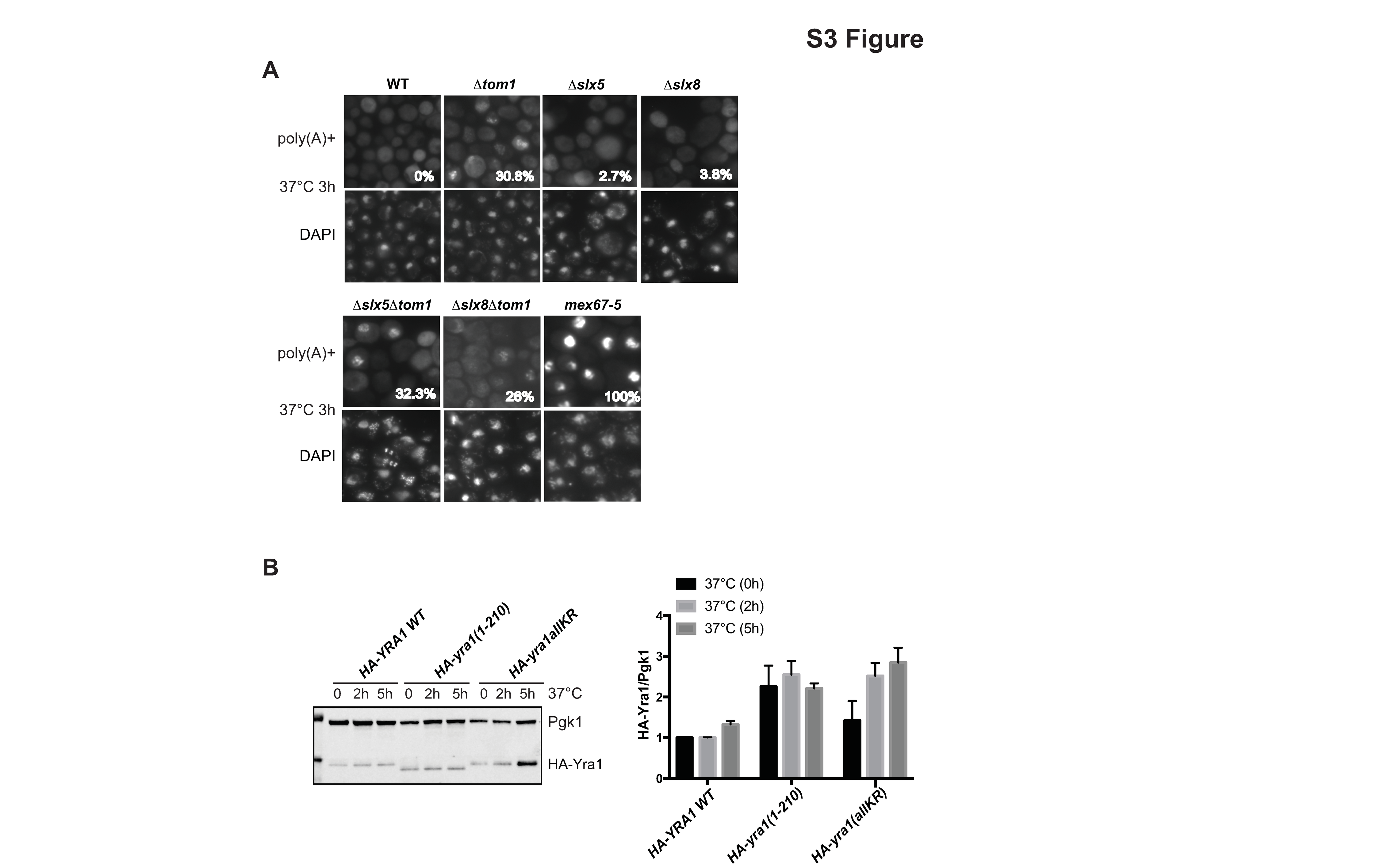

Supplement: S3 Fig — (A) Fluorescent in situ hybridization (FISH) analysis of poly(A)+ RNA localization using oligo(dT) probes on shuffled HA-YRA1 WT in WT, Δtom1, Δslx8, Δslx5, Δslx8Δtom1, Δslx5Δtom1 background and mex67-5 cells as control for mRNA export defect. The percent of cells showing poly(A)+ RNA accumulation in the nucleus is indicated in each panel. DAPI stains the cell nucleus. B) Left: Western Blot analysis of HA-Yra1 WT, HA-yra1(1–210), HA-Yra1allKR mutants grown at 25°C until exponential phase (37°C 0h) and shifted to 37°C for 2h and 5h. Right: graph representing the ratio HA-Yra1/Pgk1 of three independent experiments performed as described in Fig 2. (TIF) [file pone.0206336.s003.tif]

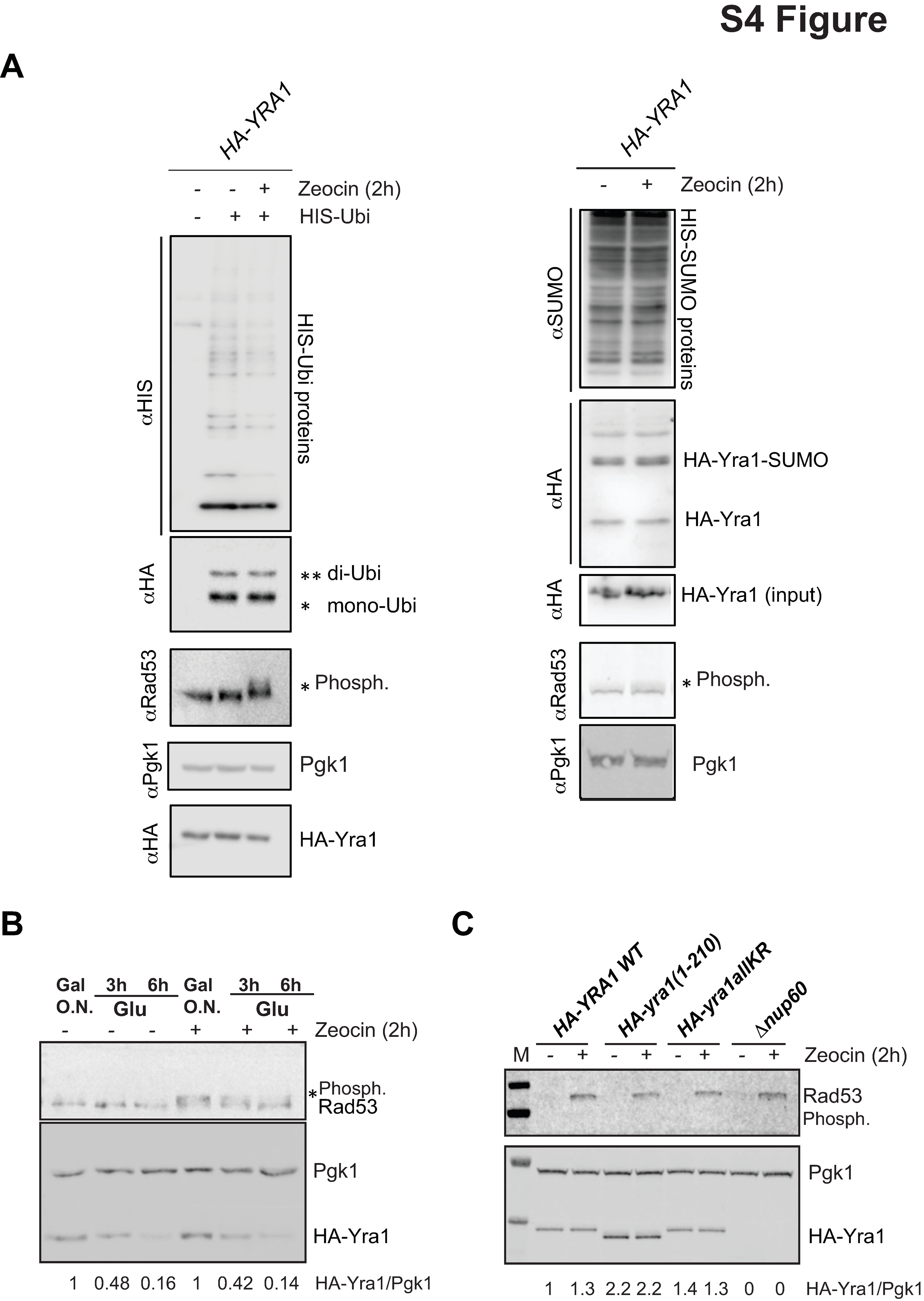

Supplement: S4 Fig — (A) Yra1 ubiquitination and sumoylation do not change under Zeocin treatment. Exponentially growing cells were treated with Zeocin (100 μg/ml) for 2h and processed for the Ubiquitination (left) and Sumoylation (right) assays as described in Materials and Methods. Rad53 phosphorylation under Zeocin treatment was revealed by Western Blot using the EL7.E1 antibody against Rad53 total protein. (B) Yra1 stability does not change under Zeocin treatment. Protein stability assay using metabolic depletion of GAL-HA-YRA1 was performed as described in Materials and Methods taking time points before (Gal O.N.) or 3h and 6h after adding Glucose. The Zeocin treatment (100 μg/ml) was started 2h before each sample collection. Rad53 phosphorylation under Zeocin was revealed by Western Blot using the EL7.E1 antibody against Rad53 total protein. Western Blot analysis of HA-Yra1 was performed as described in Materials and Methods. Quantification of the mean HA-Yra1/Pgk1 ratio of two experiments is indicated below. (C) Yra1 protein levels do not change under Zeocin treatment in the HA-YRA1 WT, HA-yra1(1–210) and HA-yra1allKR mutants. Exponentially growing cells were treated or not with Zeocin for 2h (100 μg/ml). Western blot analysis was performed as described in Materials and Methods. Quantification of the mean HA-Yra1/Pgk1 ratio of two experiments is indicated below. Rad53 phosphorylation under Zeocin treatment was revealed by Western Blot using the F9.A1 antibody against the Rad53 phosphorylated protein. (TIF) [file pone.0206336.s004.tif]

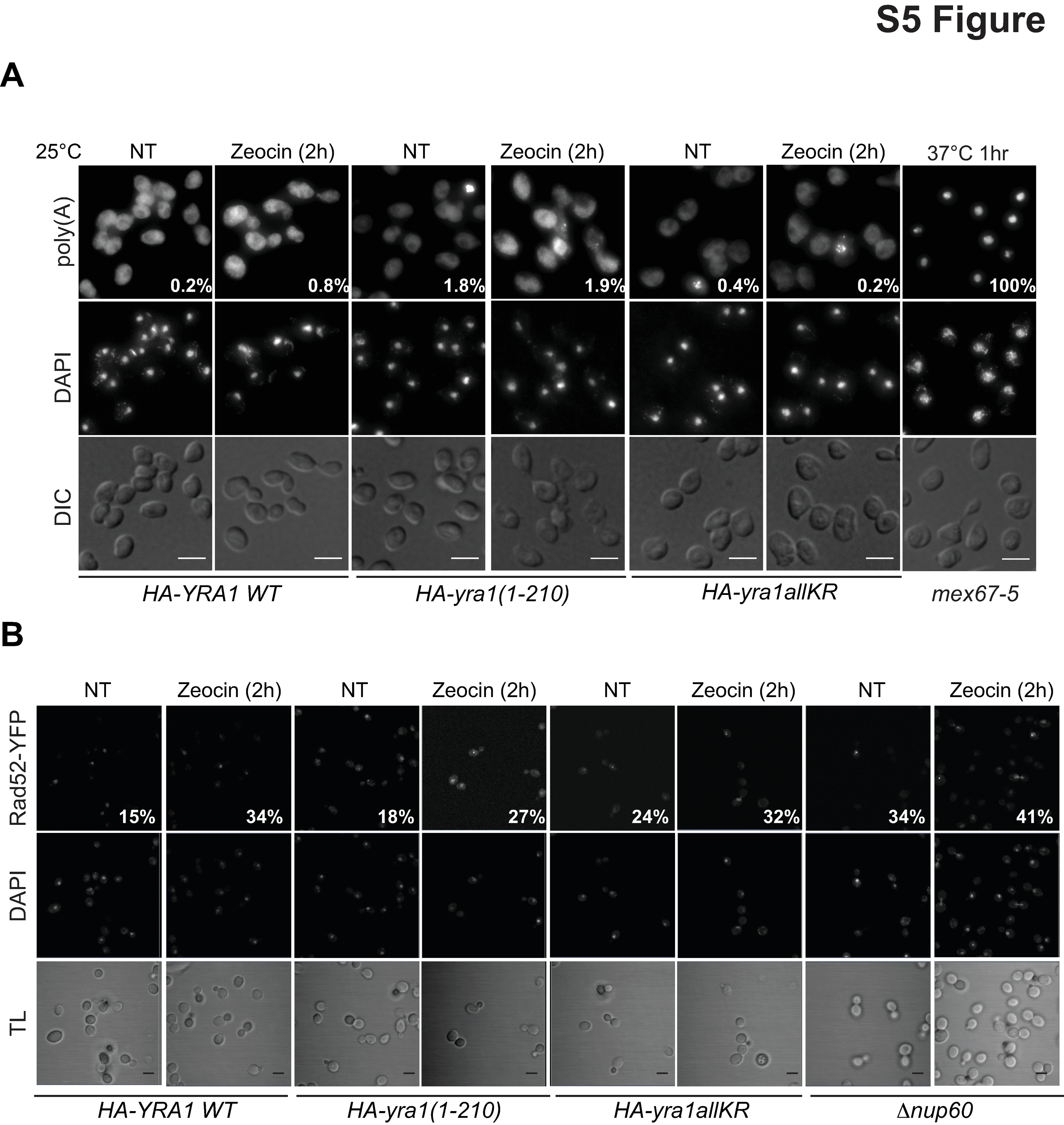

Supplement: S5 Fig — (A) HA-YRA1 WT, HA-yra1(1–210) and HA-yra1allKR have no mRNA export defect at 25°C and under Zeocin treatment. Fluorescent in situ hybridization (FISH) analysis of poly(A)+ RNA localization using oligo(dT) probes of integrated HA-YRA1 WT, HA-yra1(1–210), HA-yra1allKR and mex67-5 cells. Cells were grown exponentially in YEPD 2% Glu at 25°C and treated for 2h with Zeocin (100 μg/ml). The mex67-5 ts mutant was grown for an additional 1h at 37°C. One representative image of nuclear staining (DAPI), oligo-dT Cy3 (poly(A)+ RNA), and DIC is shown for each strain analyzed. The percent of cells showing poly(A)+ RNA accumulation in the nucleus is indicated on each panel. (B) HA-YRA1 WT, HA-yra1(1–210) and HA-yra1allKR are able to form Rad52 foci. Rad52 foci were quantified after Zeocin treatment (2h 100 μg/ml) or not (Not Treated, NT) in the indicated strains as well as in Δnup60 used as a positive control for Rad52 foci accumulation. One representative Z stack of YFP (Rad52-YFP), DAPI (nuclear staining) and Transmission Light (TL) is shown for each strain analyzed. The percent of cells with Rad52 foci is indicated in each panel. (TIF) [file pone.0206336.s005.tif]

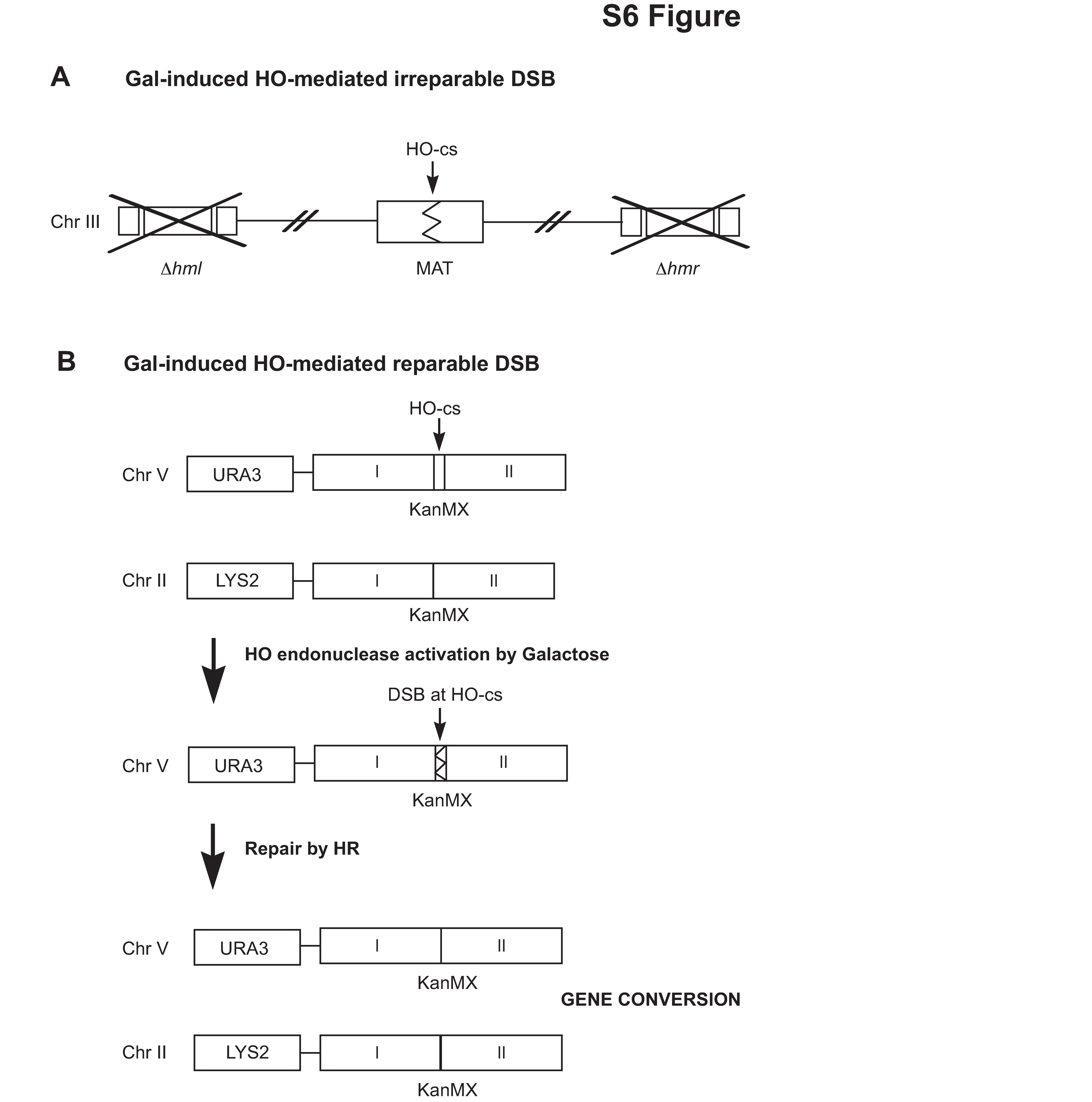

Supplement: S6 Fig — (A) Scheme showing the Gal-induced HO-mediated irreparable DSB described in [24]. The HO endonuclease is expressed in the presence of Galactose, inducing the HO cut at the Mat locus that cannot be repaired because of the deletion of HML and HMR. (B) Scheme showing the Gal-induced HO-mediated reparable DSB described in [25]. The HO endonuclease is expressed in the presence of Galactose, inducing the HO cut at the KanMx cassette next to the URA3 locus. The repair of the DSB at the HO cut is possible by HR thanks to the KanMX cassette at the LYS2 locus. If this occurs, the repair will result in an HO insensitive KanMX cassette at the URA3 locus as well as the loss of the short unique sequence surrounding the initial HO cut site. (TIF) [file pone.0206336.s006.tif]

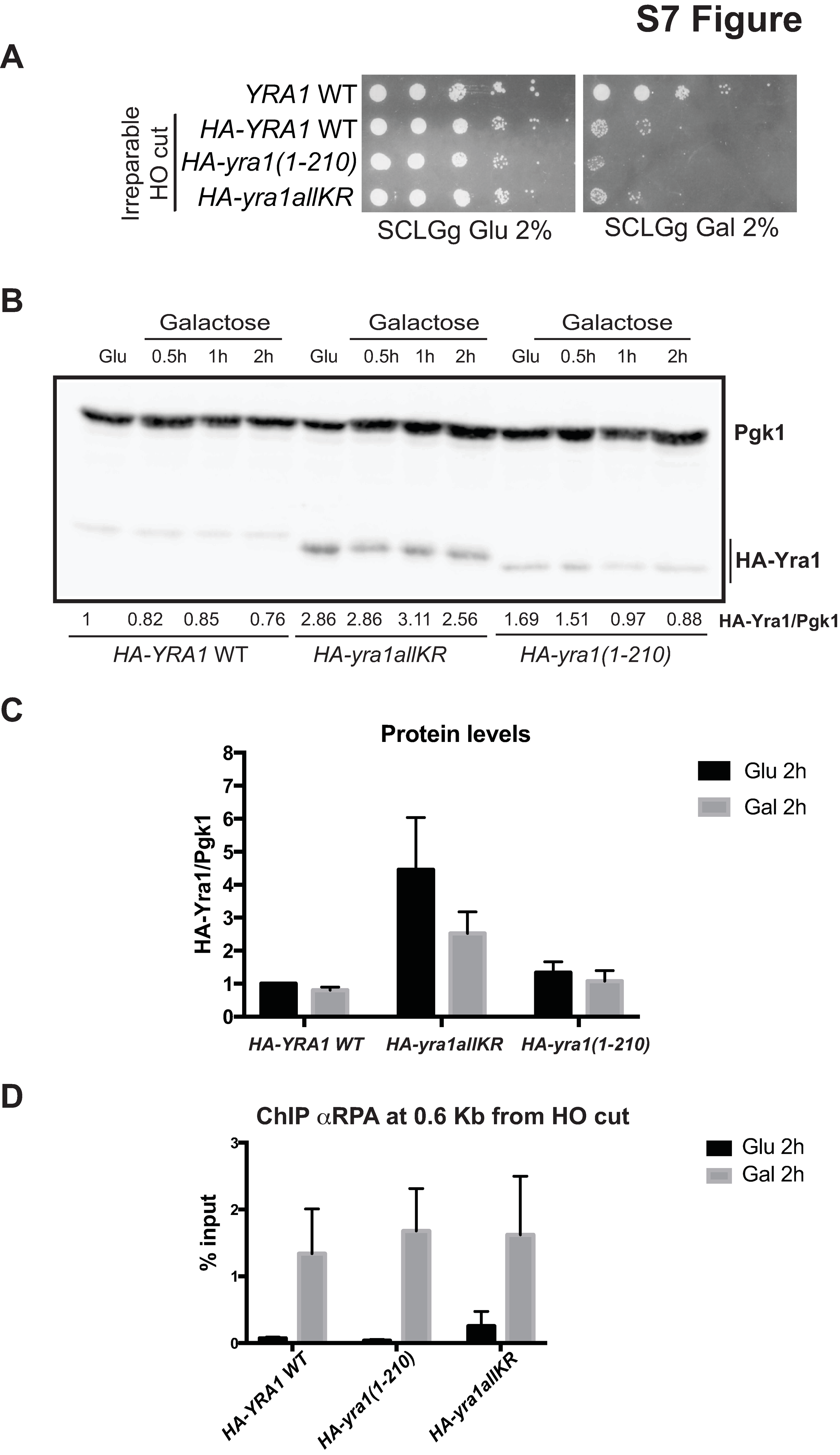

Supplement: S7 Fig — (A) Spot test analysis on plates containing SCLGg Glu 2% and SCLGg Gal 2% of confluent cultures of integrated HA-YRA1 WT and HA-yra1 mutants containing the HO irreparable DSB. A YRA1 WT strain without any galactose-inducible irreparable HO cut is shown as control. (B) Protein levels of HA-Yra1 WT, HA-yra1(1–210) and HA-yra1allKR expressed from copies integrated into the GA-6844 strain [22] after 2h in Glucose or Galactose to induce the irreparable HO cut. The levels of WT or mutant HA-Yra1 proteins remain quite constant between the different time points Glu 2h and Gal (0.5h, 1h, 2h). Values of HA-Yra1/Pgk1 are shown below the blot. One representative Western Blot is shown. (C) Quantification of the Western blot. The average of 3 independent experiments is shown with corresponding standard error of the mean. (D) RPA recruitment to the HO cut site in yra1 mutants. ChIP using αRPA antibody of HA-YRA1 WT (WT), HA-yra1(1–210) and HA-yra1allKR, at 0.6 Kb from the HO cut site after 2h of HO induction with Galactose. The 2h Glucose time point was taken as no cut control. ChIP values are shown as percentage of input. The average of 3 independent experiments is shown with corresponding standard error of the mean. (TIF) [file pone.0206336.s007.tif]

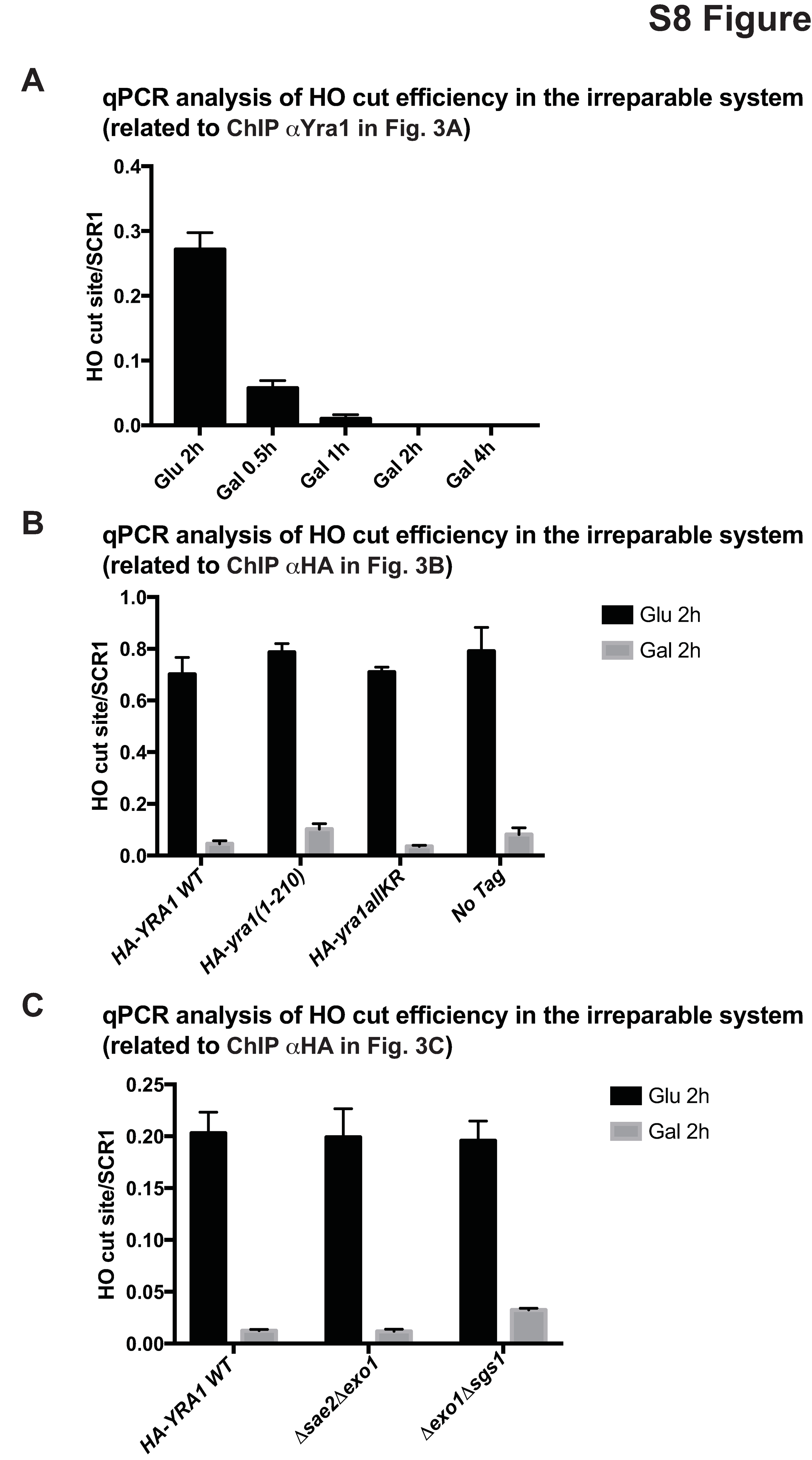

Supplement: S8 Fig — (A) Analysis of HO cut site levels in the GA6844 strain described in [22] after 0.5h, 1h, 2h and 4h of HO endonuclease induction with galactose. The HO cut genomic locus was quantified by qPCR and the level was normalized to SCR1. The average of 6 independent experiments is shown with corresponding standard error of the mean. (B) Analysis of HO cut site levels in the HA-YRA1 WT and HA-yra1 mutants integrated in GA6844 strain described in [22] after 2h of HO endonuclease induction with galactose or 2h in Glucose (no HO induction). The average of 3 independent experiments is shown with corresponding standard error of the mean. (C) Analysis of HO cut site levels in the HA-YRA1 WT and Δsae2Δexo1, Δexo1Δsgs1 mutants. The average of 4 independent experiments is shown with corresponding standard error of the mean. (TIF) [file pone.0206336.s008.tif]

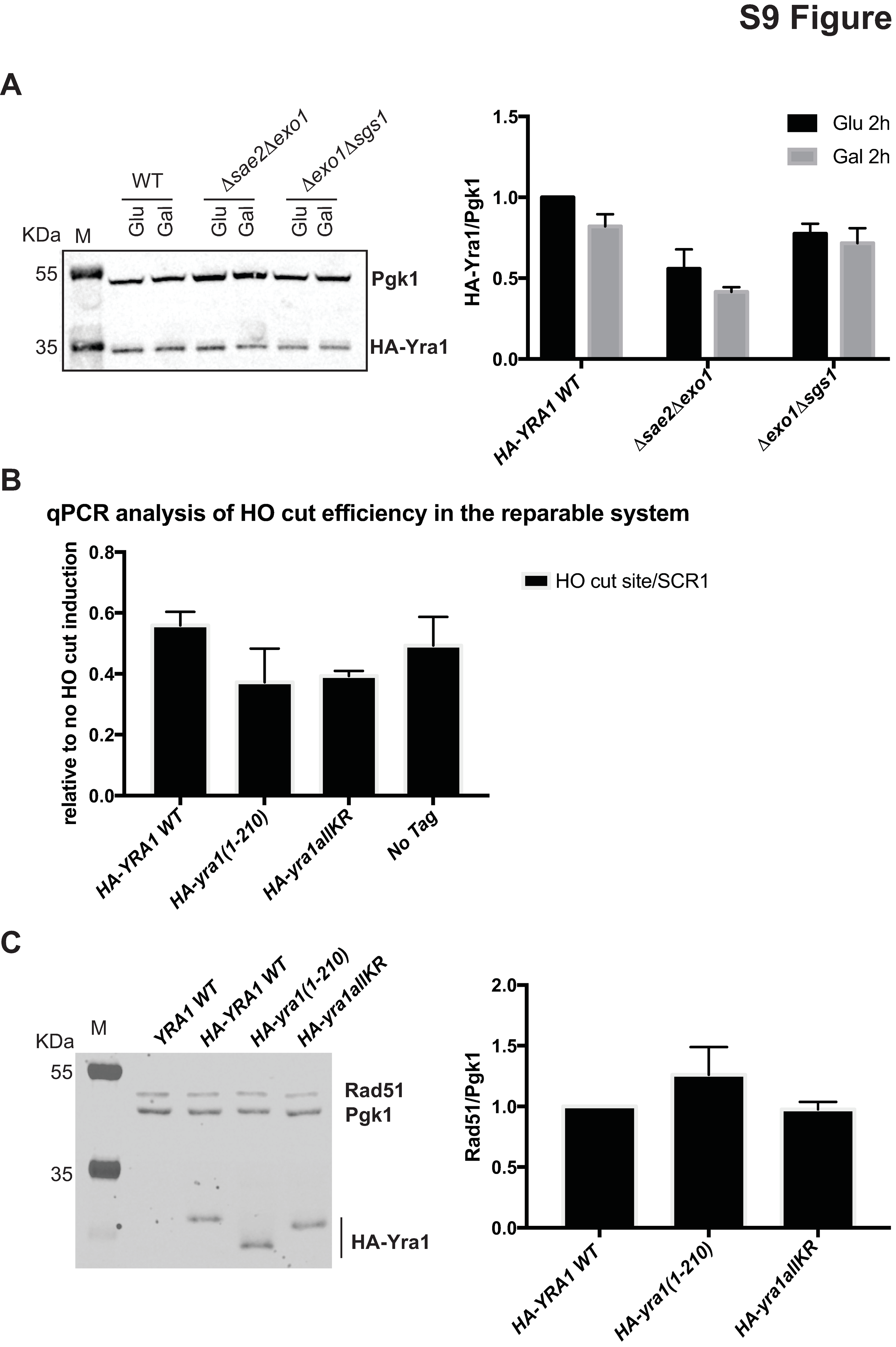

Supplement: S9 Fig — (A) Left: Western Blot analysis related to Fig 3C of HA-Yra1 in WT, Δsae2Δexo1, Δexo1Δsgs1 mutants and performed as described in Materials and Methods. Right: Quantification and average of 4 experiments with corresponding standard error of the mean. (B) Analysis of HO cut site levels in HA-YRA1 WT (WT), HA-yra1(1–210), HA-yra1allKR and No-Tag strains treated with Galactose 2% (cut induction) or not (control) for 2h. The average of 2 independent experiments is shown with corresponding standard error of the mean. (C) Levels of Rad51 in HA-YRA1 WT and HA-yra1 mutant strains analyzed by Western blot. Right: Western blot quantification of the ratio of Rad51/Pgk1 of three independent experiments with relative standard error of the mean. (TIF) [file pone.0206336.s009.tif]
