# Supplementary material for: The mRNA export adaptor Yra1 contributes to DNA double-strand break repair through its C-box domain
Source: PLoS One. 2019 Apr 5;14(4):e0206336. doi: 10.1371/journal.pone.0206336 (PMC6450643; doi:10.1371/journal.pone.0206336)
Supplement: S1 Table — (DOCX) [file pone.0206336.s010.docx]

# S1 Table: Strains used in this study

| **Strains** | **Name** | **Genotype** | **Reference** |
| --- | --- | --- | --- |
| **FSY1026** | *YRA1 shuffle* | *MATa ade2 leu2 trp1 ura3 ∆yra1::HIS3+ <YCplac33 URA3 YRA1>* | [1] |
| **FSY1188** | *HA-YRA1 WT shuffled* | *MATa ade2 leu2 trp1 ura3 ∆yra1::HIS3+ <YCplac22 TRP1 HA-YRA1 WT >* | This study |
| **FSY4976** | *WT (W303)* | *MATa ade2 leu2 his3 trp1 ura3* | EUROSCARF |
| **FSY4726** | *Δslx8 YRA1 shuffle* | *MATa ade2 leu2 trp1 ura3 ∆yra1::HIS3+ <YCplac33 URA3 YRA1>, Δslx8::LEU2* | This study |
| **FSY4917** | *Δslx5 YRA1 shuffle* | *MATa ade2 leu2 trp1 ura3 ∆yra1::HIS3+ <YCplac33 URA3 YRA1>, Δslx5::KANr* | This study |
| **FSY4934** | *Δslx5, Δslx8 YRA1 shuffle* | *MATa ade2 leu2 trp1 ura3 ∆yra1::HIS3+ <YCplac33 URA3 YRA1>, Δslx5::KANr, Δslx8::LEU2* | This study |
| **FSY4935** | *Δtom1, Δslx5 YRA1 shuffle* | *MATa ade2 leu2 trp1 ura3 ∆yra1::HIS3+ <YCplac33 URA3 YRA1>, Δtom1::KANr, Δslx5::KANr* | This study |
| **FSY4936** | *Δtom1, Δslx8 YRA1 shuffle* | *MATa ade2 leu2 trp1 ura3 ∆yra1::HIS3+ <YCplac33 URA3 YRA1>, Δtom1::KANr, Δslx8::LEU2* | This study |
| **FSY3373** | *Δtom1 YRA1 shuffle* | *MATa ade2 leu2 trp1 ura3 ∆yra1::HIS3+ <YCplac33 URA3 YRA1>, Δtom1::KANr* | [2] |
| **FSY3412** | *Δtom1 HA YRA1 WT shuffled* | *MATa ade2 leu2 trp1 ura3 ∆yra1::HIS3+ <YCplac22 TRP1 HA-YRA1WT>, Δtom1::KANr* | This study |
| **FSY4753** | *Δslx8 HA YRA1 WT shuffled* | *MATa ade2 leu2 trp1 ura3 ∆yra1::HIS3+ <YCplac22 TRP1 HA-YRA1WT>, Δslx8::LEU2* | This study |
| **FSY4937** | *Δslx5 HA YRA1 WT shuffled* | *MATa ade2 leu2 trp1 ura3 ∆yra1::HIS3+ <YCplac22 TRP1 HA-YRA1WT>, Δslx5::KANr* | This study |
| **FSY4938** | *Δslx5, Δslx8 HA YRA1 WT shuffled* | *MATa ade2 leu2 trp1 ura3 ∆yra1::HIS3+ <YCplac22 TRP1 HA-YRA1WT>, Δslx5::KANr, Δslx8::LEU2* | This study |
| **FSY4939** | *Δtom1, Δslx5 HA YRA1 WT shuffled* | *MATa ade2 leu2 trp1 ura3 ∆yra1::HIS3+ <YCplac22 TRP1 HA-YRA1WT>, Δtom1::KANr, Δslx5::KANr* | This study |
| **FSY4941** | *Δtom1, Δslx8 HA YRA1 WT shuffled* | *MATa ade2 leu2 trp1 ura3 ∆yra1::HIS3+ <YCplac22 TRP1 HA-YRA1WT>, Δtom1::KANr, Δslx8::LEU2* | This study |
| **FSY50410** | *Δsiz1 (YV1168)* | *MATa ade2 leu2 trp1 ura3 his3 RAD5 can1 Δsiz1::KANr* | B. Palancade |
| **FSY5051** | *Δsiz2 (YV1169)* | *MATa ade2 leu2 trp1 ura3 his3 RAD5 can1 Δsiz2::KANr* | B. Palancade |
| **FSY5052** | *Δsiz1,Δsiz2 (YV1077)* | *MATa ade2 leu2 trp1 ura3 his3 RAD5 can1 Δsiz1::KANr, Δsiz2::KANr* | B. Palancade |
| **FSY5053** | *mms21-11 (YV1084)* | *MATa ade2 leu2 trp1 ura3 his3 RAD5 can1 mms21-11::KANr* | B. Palancade |
| **FSY3992** | *ulp1 ts* | *MATa ade2 leu2 trp1 ura3 ∆ulp1::HIS3+ <YCplac22 TRP1 ulp1-ts>* | This study |
| **FSY7017** | *HA-YRA1 WT integrated* | *MATa ade2 leu2 his3 trp1 ura3 HA-YRA1WT::HIS5* | This study |
| **FSY7019** | *HA-yra1(1-210) integrated* | *MATa ade2 leu2 his3 trp1 ura3 HA-yra1(1-210)::HIS5* | This study |
| **FSY7022** | *HA-yra1allKR integrated* | *MATa ade2 leu2 his3 trp1 ura3 HA-yra1allKR::HIS5* | This study |
| **FSY7158** | *Δrad52, HA-YRA1 WT integrated* | *MATa ade2 leu2 his3 trp1 HA-YRA1WT::HIS5, Δrad52::NATr* | This study |
| **FSY1982** | *mex67-5* | *MATa ade2 his3 leu2 trp1 ura3 mex67-5 integrated* | [3] |
| **FSY5073** | *GA-6844* | *JKM179, MATα, Δhml::ADE1 hmr::ADE1 ade3::GALHO ade1-100 leu2-3, 112 lys5 trp1::hisG ura3-52CFP-NUP49 GFP-LacI:Leu2 MAT::LacO repeats:TRP1* | [4] |
| **FSY6286** | *HA-YRA1 WT integrated in GA-6844* | *JKM179, MATα, Δhml::ADE1 hmr::ADE1 ade3::GALHO ade1-100 leu2-3, 112 lys5 trp1::hisG ura3-52CFP-NUP49 GFP-LacI:Leu2 MAT::LacO repeats:TRP1, HA-YRA1WT::URA3* | This study |
| **FSY6287** | *HA-yra1(1-120) integrated in GA-6844* | *JKM179, MATα, Δhml::ADE1 hmr::ADE1 ade3::GALHO ade1-100 leu2-3, 112 lys5 trp1::hisG ura3-52CFP-NUP49 GFP-LacI:Leu2 MAT::LacO repeats:TRP1, HA-yra1(1-210)::URA3* | This study |
| **FSY6288** | *HA-yra1allKR integrated in GA-6844* | *JKM179, MATα, Δhml::ADE1 hmr::ADE1 ade3::GALHO ade1-100 leu2-3, 112 lys5 trp1::hisG ura3-52CFP-NUP49 GFP-LacI:Leu2 MAT::LacO repeats:TRP1, HA-yra1allKR::URA3* | This study |
| **FSY6881** | *NA17* | *MK225, MATa-inc, ade3::GALHO ade2-1 leu2-3, 112 his3-11,15 trp1-1 can1-100,KanMX::HO-cs in URA3, KanMX::ClaI in LYS2* | [5] |
| **FSY7181** | *HA-YRA1 WT integrated in NA17* | *MK225, MATa-inc, ade2-1 leu2-3, 112 his3-11,15 trp1-1 can1-100,KanMX::HO-cs in URA3, KanMX::ClaI in LYS2, HA-YRA1WT::HIS5* | This study |
| **FSY7183** | *HA-yra1(1-120) integrated in NA17* | *MK225, MATa-inc, ade2-1 leu2-3, 112 his3-11,15 trp1-1 can1-100,KanMX::HO-cs in URA3, KanMX::ClaI in LYS2, HA-yra1(1-210)::HIS5* | This study |
| **FSY7186** | *HA-yra1allKR integrated in NA17* | *MK225, MATa-inc, ade2-1 leu2-3, 112 his3-11,15 trp1-1 can1-100,KanMX::HO-cs in URA3, KanMX::ClaI in LYS2, HA-yra1allKR::HIS5* | This study |
| **FSY7738** | *Δrad52 integrated in NA17* | *MK225, MATa-inc, ade3::GALHO ade2-1 leu2-3, 112 his3-11,15 trp1-1 can1-100,KanMX::HO-cs in URA3, KanMX::ClaI in LYS2,Δrad52::NATr* | This study |
| **FSY8346** | *HA-YRA1 WT in GA6844, Δsae2, Δexo1* | *JKM179, MATα, Δhml::ADE1 hmr::ADE1 ade3::GALHO ade1-100 leu2-3, 112 lys5 trp1::hisG ura3-52CFP-NUP49 GFP-LacI:Leu2 MAT::LacO repeats:TRP1, HA-YRA1WT::URA3, Δsae2::Kan^r^, Δexo1::NAT^r^* | This study |
| **FSY8347** | *HA-yra1(1-120) in GA6844, Δsae2, Δexo1* | *JKM179, MATα, Δhml::ADE1 hmr::ADE1 ade3::GALHO ade1-100 leu2-3, 112 lys5 trp1::hisG ura3-52CFP-NUP49 GFP-LacI:Leu2 MAT::LacO repeats:TRP1, HA-yra1(1-210)::URA3, Δsae2::Kan^r^, Δexo1::NAT^r^* | This study |
| **FSY8348** | *HA-yra1allKR in GA6844, Δsae2, Δexo1* | *JKM179, MATα, Δhml::ADE1 hmr::ADE1 ade3::GALHO ade1-100 leu2-3, 112 lys5 trp1::hisG ura3-52CFP-NUP49 GFP-LacI:Leu2 MAT::LacO repeats:TRP1, HA-yra1allKR::URA3, Δsae2::Kan^r^, Δexo1::NAT^r^* | This study |
| **FSY8351** | *HA-YRA1 WT in GA6844, Δsgs1, Δexo1* | *JKM179, MATα, Δhml::ADE1 hmr::ADE1 ade3::GALHO ade1-100 leu2-3, 112 lys5 trp1::hisG ura3-52CFP-NUP49 GFP-LacI:Leu2 MAT::LacO repeats:TRP1, HA-YRA1WT::URA3, Δsgs1::Kan^r^, Δexo1::NAT^r^* | This study |

# References

1. Zenklusen D, Vinciguerra P, Strahm Y, Stutz F. The yeast hnRNP-Like proteins Yra1p and Yra2p participate in mRNA export through interaction with Mex67p. Mol Cell Biol. 2001;21(13):4219-32.

2. Iglesias N, Tutucci E, Gwizdek C, Vinciguerra P, Von Dach E, Corbett AH, et al. Ubiquitin-mediated mRNP dynamics and surveillance prior to budding yeast mRNA export. Genes Dev. 2010;24(17):1927-38.

3. Jimeno S, Rondon AG, Luna R, Aguilera A. The yeast THO complex and mRNA export factors link RNA metabolism with transcription and genome instability. EMBO J. 2002;21(13):3526-35.

4. Horigome C, Oma Y, Konishi T, Schmid R, Marcomini I, Hauer MH, et al. SWR1 and INO80 chromatin remodelers contribute to DNA double-strand break perinuclear anchorage site choice. Mol Cell. 2014;55(4):626-39.

5. Agmon N, Liefshitz B, Zimmer C, Fabre E, Kupiec M. Effect of nuclear architecture on the efficiency of double-strand break repair. Nat Cell Biol. 2013;15(6):694-9.
