# Supplementary material for: The mRNA export adaptor Yra1 contributes to DNA double-strand break repair through its C-box domain
Source: PLoS One. 2019 Apr 5;14(4):e0206336. doi: 10.1371/journal.pone.0206336 (PMC6450643; doi:10.1371/journal.pone.0206336)
Supplement: S2 Table — (DOCX) [file pone.0206336.s011.docx]

# S2 Table: Plasmids used in this study

| **Code** | **Name** | **Description** | **Reference** |
| --- | --- | --- | --- |
| **pFS 3087** | *pRS426 His6-UBI* | *Ubiquitin under CUP1 promoter (URA3, 2μ)* | [1] |
| **pFS 3571** | *pRS426 empty* | *Empty vector (URA3, 2μ)* |  |
| **pFS 3613** | *pRS426 His6-SMT3* | *SUMO under CUP1 promoter (URA3, 2μ)* | *B. Palancade* |
| **pFS 3688** | *YCpLac111 LEU2 GAL1p HA-YRA1 WT* | *YRA1 cloned as SalI fragment into pFS3647 (Gal1p HA-SalI-+500 YRA1)* | *This study* |
| **pFS 3687** | *YCpLac22 TRP1 GAL1p HA-YRA1 WT* | *YRA1 cloned as HindIII-SpeI from pFS3688 into pFS2233 (TRP1, CEN)* | *This study* |
| **pFS 1341** | *YCpLac111 LEU2* | *Empty vector (LEU2, CEN)* |  |
| **pFS 3932** | *pUC18 URA3 HA-YRA1 WT* | *pUC18 with SmaI fragment containing 500 bp YRA1 5' flanks, an ATG and HA-YRA1 WT followed by 500bp Yra1 3' flanks, URA3 marker and UTR-YDR381C sequence* | *This study* |
| **pFS 3934** | *pUC18 URA3 HA-yra1(1-210)* | *pUC18 with SmaI fragment containing 500 bp YRA1 5' flanks, an ATG and HA-yra1(1-210) followed by 500bp Yra1 3' flanks, URA3 marker and UTR-YDR381C sequence* | *This study* |
| **pFS 3933** | *pUC18 URA3 HA-yra1allKR* | *pUC18 with SmaI fragment containing 500 bp YRA1 5' flanks, an ATG and HA-yra1allKR followed by 500bp Yra1 3' flanks, URA3 marker and UTR-YDR381C sequence* | *This study* |
| **pFS 4131** | *pRS415 pGAL1-HO LEU2* | *HO endonuclease under GAL1 promoter* | *David Shore Lab* |
| **pFS 4082** | *pUC18 HIS5 HA-YRA1 WT* | *pUC18 with SmaI fragment containing 500 bp YRA1 5' flanks, an ATG and HA-YRA1 WT followed by 500bp Yra1 3' flanks, HIS5 marker and UTR-YDR381C sequence* | *This study* |
| **pFS 4083** | *pUC18 HIS5 HA-yra1(1-210)* | *pUC18 with SmaI fragment containing 500 bp YRA1 5' flanks, an ATG and HA-yra1(1-210) followed by 500bp Yra1 3' flanks, HIS5 marker and UTR-YDR381C sequence* | *This study* |
| **pFS 4085** | *pUC18 HIS5 HA-yra1allKR* | *pUC18 with SmaI fragment containing 500 bp YRA1 5' flanks, an ATG and HA-yra1allKR followed by 500bp Yra1 3' flanks, HIS5 marker and UTR-YDR381C sequence* | *This study* |
| **pFS 3118** | *pUG27 HIS5* | *Empty vector (HIS5)* |  |
| **pFS 3574** | *pUC18* |  |  |
| **pFS 3864** | *YCpLac111 LEU2 Rad52-YFP* | *Rad52-YFP cloned as SalI fragment in*  *YCpLac111 LEU2, CEN* | *This study* |

# References

1. Vitaliano-Prunier A, Menant A, Hobeika M, Geli V, Gwizdek C, Dargemont C. Ubiquitylation of the COMPASS component Swd2 links H2B ubiquitylation to H3K4 trimethylation. Nat Cell Biol. 2008;10(11):1365-71.
