# Supplementary material for: The mRNA export adaptor Yra1 contributes to DNA double-strand break repair through its C-box domain
Source: PLoS One. 2019 Apr 5;14(4):e0206336. doi: 10.1371/journal.pone.0206336 (PMC6450643; doi:10.1371/journal.pone.0206336)
Supplement: S3 Table — (DOCX) [file pone.0206336.s012.docx]

# S3 Table: Primers used in this study

| **Code** | **SEQUENCE** | **Description** | **Reference** |
| --- | --- | --- | --- |
| **OFS 1840** | GGGGCGGCCAAGTAACAAACGAGTAAATTAATTACACCTTGTcagctgaagcttcgtacgc | *SLX8* deletion Fwd | *This study* |
| **OFS 1841** | CCCGGCTACTCCATAGAATCTTCTTTTGAGTATGCCTGTAAAACGgcataggccactagtggatctg | *SLX8* deletion Rev | *This study* |
| **OFS 1869** | TTATTATTTGGAACGCGGAGCTCCTCTAATAGTCGAAATAATAGAcggatccccgggttaattaa | *SLX5* deletion Fwd | *This study* |
| **OFS 1870** | TGATGATAAGTTCGAAAATGCCCTTATAAAAATTAAACCGCGTGgaattcgagctcgtttaaac | *SLX5* deletion Rev | *This study* |
| **OFS 2670 (SG-1879)** | TTGCCCACTTCTAAGCTGATTTC | 0.6 Kb from HO site in Matα Fwd | [1] |
| **OFS 2671 (SG-1880)** | GTACTTTTCTACATTGGGAAGCAATAAA | 0.6 Kb from HO site in Matα Rev | [1] |
| **OFS 2672 (SG-573)** | GTTCTCATGCTGTCGAGGATTTT | 1.6 Kb from HO site in Matα Fwd | [1] |
| **OFS 2673 (SG-574)** | AGACGTCCTTCTACAACAATTCATAAGT | 1.6 Kb from HO site in Matα Rev | [1] |
| **OFS 2674 (SG-1883)** | AACTGGCAAAGGTCTATGTAAAGATTTA | 4.5 Kb from HO site in Matα Fwd | [1] |
| **OFS 2675 (SG-1884)** | AATGGATGAAGATGATGACGTTGAC | 4.5 Kb from HO site in Matα Rev | [1] |
| **OFS 2676 (SG-1885)** | CGTGGTTATGTATTGGTACTATTTCTTG | 9.6 Kb from HO site in Matα Fwd | [1] |
| **OFS 2677 (SG-1886)** | AATTGGATAATTTGAAATCTGGTAACCC | 9.6 Kb from HO site in Matα Rev | [1] |
| **OFS 2678 (SG-1887)** | TCTTAACGTGAACGGCAGTGA | 23 Kb from HO site in Matα Fwd | [1] |
| **OFS 2679 (SG-1888)** | TGAATCTTCTCCATACGCTGCTAT | 23 Kb from HO site in Matα Rev | [1] |
| **OFS 2682**  **(SG-2285)** | AATATGGGACTACTTCGCGCAACA | HO cut efficiency Fwd | [1] |
| **OFS 2683**  **(SG-2286)** | CGTCACCACGTACTTCAGCATAA | HO cut efficiency Rev | [1] |
| **OFS 2798** | TAGTGCATATTTAGTTTACTTTTTGCCTTTGATTGAAAATATATATTCcggatccccgggttaattaa | *TOM1* deletion Fwd | *This study* |
| **OFS 2799** | CGTTCTAAAATACTTGGTTACATGGCGCTATAAATTTACACGAAAAATGACGATGAATTCGAGCTCGTTT | *TOM1* deletion Rev | *This study* |
| **OFS 2790** | aggtcgactctagaggatccccgggTACCACTACCACAGAGTTCTTTG | Fwd fragment 1 Gibson assembly pUC18-SmaI (-314) YRA1 | *This study* |
| **OFS 2791** | **GCAGCGTACGAAGCT**CGTCACCGATGAGTAGGTTA | Rev fragment 1 Gibson assembly (+183) YRA1-pUG | *This study* |
| **OFS 2792** | TAACCTACTCATCGGTGACGagcttcgtacgctgc | Fwd fragment 2 Gibson assembly 3' YRA1-pUG | *This study* |
| **OFS 2793** | GTCAAATATGCCGAATAAACcataggccactagtggatctg | Rev fragment 2 Gibson assembly pUG-3' YRA1 | *This study* |
| **OFS 2794** | **CAGATCCACTAGTGGCCTATG**GTTTATTCGGCATATTTGAC | Fwd fragment 3 Gibson assembly pUG- 3' YDR381 | *This study* |
| **OFS 2795** | acgaattcgagctcggtacccggggCATTCTTTGAGCCGTACT | Rev fragment 3 Gibson assembly 5' YDR381-SmaI-pUC18 | *This study* |
| **OFS 2916** | GCAAACAAGGAGGTTGCCAAGAACTGCTGAAGGTTCTGGTGGCTTTGGTGTGTTGTTGcggatccccgggttaattaa | *RAD52* deletion Fwd | *This study* |
| **OFS 2917** | AGGATTTTGGAGTAATAAATAATGATGCAAATTTTTTATTTGTTTCGGCCAGGAAGCGTTCGATGAATTCGAGCTCGTTT | *RAD52* deletion Rev | *This study* |
| **OFS 1717** | AACCGTCTTTCCTCCGTCGTAA | *SCR1* Fwd qPCR | *This study* |
| **OFS 1718** | CTACCTTGCCGCACCAGACA | *SCR1* Rev qPCR | *This study* |
| **OFS 4086** | CAGCCAGTTTAGTCTGACCA | KAN IV Rev | *This study* |
| **OFS 4088 (OI3)** | GTACGGTACCACTGAAACACAGCGTGCAG | Fwd on *LYS2* LOCUS used with OFS4086 to check KanMX::ClaI | [2] |
| **OFS 4090 (OI9)** | TTTTGCGAGGCATATTTATGGTGAAGG | Fwd on *URA3* LOCUS used with OFS4086 to check KanMX::HO | [2] |
| **OFS 4188** | GAATTTCAGCTTTCCGCAA | HO site in NA17 background Fwd qPCR | *This study* |
| **OFS 4179/A** | GGTATTCTGGGCCTCCATGT | HO site in NA17 background Rev qPCR | *This study* |
| **OFS 3118** | GCACTCTCATTCAATGTCC | Fwd -600 *YRA1* | *This study* |
| **OFS 3120** | GTAGTCTGGGACGTCGTATG | Rev HA tag | *This study* |

# References

1. Horigome C, Oma Y, Konishi T, Schmid R, Marcomini I, Hauer MH, et al. SWR1 and INO80 chromatin remodelers contribute to DNA double-strand break perinuclear anchorage site choice. Mol Cell. 2014;55(4):626-39.

2. Agmon N, Liefshitz B, Zimmer C, Fabre E, Kupiec M. Effect of nuclear architecture on the efficiency of double-strand break repair. Nat Cell Biol. 2013;15(6):694-9.
